# M-ECG: extracting heart signals with a novel computational analysis of magnetoencephalography data

**DOI:** 10.3389/fnimg.2025.1675960

**Published:** 2026-01-09

**Authors:** Aqil Izadysadr, Hamideh Sadat Bagherzadeh, Jennifer R. Stapleton-Kotloski, Gautam S. Popli, Cormac A. O'Donovan, Dwayne W. Godwin

**Affiliations:** 1 Department of Neurology, Wake Forest School of Medicine, Winston-Salem, NC, United States; 2 Department of Translational Neuroscience, Wake Forest School of Medicine, Winston-Salem, NC, United States

**Keywords:** magnetoencephalography, MEG-derived electrocardiogram (M-ECG), heart rate variability (HRV), autonomic dysfunction, multimodal imaging

## Abstract

Magnetoencephalography (MEG) captures neural activity with high temporal and spatial resolution, but it typically discards other biopotentials, such as cardiac signals, as noise. Here, we demonstrate the feasibility of extracting cardiac signals from MEG recordings using a novel algorithm to compute heart rate variability (HRV), a key autonomic biomarker. Using the Brainstorm MEG auditory dataset and the Open MEG Archive resting-state sample dataset, we developed an approach that isolates MEG-derived electrocardiogram (M-ECG) using either independent component analysis or MEG reference sensors. This algorithm identifies physiologically valid R-peaks, removes outliers, and corrects aberrant RR intervals to enable accurate HRV computation. We evaluated HRV derived from M-ECG against HRV derived from simultaneously recorded electrocardiogram (ECG) using time-domain and frequency-domain measures, along with non-parametric statistical tests and similarity metrics. Results revealed strong temporal and spectral agreement between M-ECG and simultaneously recorded ECG signals, including alignment across HRV bands and minimal bias in RR intervals. These findings highlight the potential of M-ECG for non-invasively assessing autonomic function using existing MEG data. Incorporating HRV into MEG studies could advance our understanding of brain-heart interactions and provide new diagnostic and prognostic insights, particularly in neurological disorders involving autonomic dysregulation.

## 1 Introduction

Magnetoencephalography (MEG) is a non-invasive neurophysiological technique that measures the magnetic fields generated by neuronal activity in the brain on a sub-millisecond scale (Singh, 2014).

MEG scanners usually operate in magnetically-shielded rooms to reduce environmental noise (Vrba and Wilson, 2007; Singh, 2014). To further suppress residual noise, reference sensors such as magnetometers or gradiometers are used to capture ambient magnetic fluctuations and enable signal-space noise cancellation (Vrba and Wilson, 2007; Singh, 2014). Although these reference channels are primarily intended for noise reduction, they also record weak but biologically meaningful magnetic fields generated by physiological sources outside the brain, including cardiac and muscular activity.

Conventional MEG preprocessing removes artifacts, such as heartbeats, eye movements, and environmental noise, to improve the fidelity of neural signals (Escudero et al., 2007; Breuer et al., 2014). Independent component analysis (ICA) is one of the most widely used methods for this purpose, as it separates statistically independent sources and allows isolation of cardiac components without requiring prior knowledge of their waveform or timing (Escudero et al., 2007). While the main goal of artifact removal is to enhance brain signal quality, these cardiac and physiological components may themselves carry useful information.

In particular, heart rate variability (HRV) serves as a significant biomarker in the study of brain disorders (Arakaki et al., 2023). The autonomic nervous system regulates heart rate and important brain functions, so dysfunction of the autonomic nervous system is often associated with various neurological and psychiatric disorders, including epilepsy, Parkinson's disease, and mood disorders (Bassett, 2016; Myers et al., 2018; Arnao et al., 2020; Arakaki et al., 2023). For example, in major depressive disorder, lower vagally mediated HRV during cognitive tasks is associated with impaired inhibitory control, reflecting disrupted prefrontal connectivity and altered brain-body integration (Zhou et al., 2020). Furthermore, a range of brain disorders, including stroke, dementia, traumatic brain injury, post-traumatic stress disorder, and sudden unexpected death in epilepsy, carry elevated cardiovascular risk (Samuels, 2007; Tan et al., 2011; Baysal-Kirac et al., 2017; Deckers et al., 2017; Wolters et al., 2018; Schnabel et al., 2021; Song et al., 2021; Stewart et al., 2022). As such, HRV offers a non-invasive window into autonomic nervous system dysregulation and its role in brain pathology.

The RR interval, measured between consecutive R-waves in the electrocardiogram (ECG), is the primary marker of HRV and cardiac rhythmicity (Lanfranchi and Somers, 2011; Peltola, 2012). HRV is typically analyzed across three major bands: very-low-frequency, low-frequency, and high-frequency bands (Usui and Nishida, 2017). The very-low-frequency band typically spans frequencies from 0.0033 to 0.04 Hz, reflecting the activity of the central nervous system (Armstrong et al., 2022; McCraty, 2022); however, in short recordings such as those used in this study, spectral estimates in this band are unreliable and are therefore not interpreted here. The low-frequency band typically spans frequencies from 0.04 to 0.15 Hz, reflecting sympathetic and parasympathetic modulation (Batchinsky et al., 2007; Cabiddu et al., 2012; Duprey et al., 2019; Scavone et al., 2021), while the high-frequency band covers frequencies from 0.15 to 0.4 Hz, primarily indicating parasympathetic (vagal) activity and respiratory sinus arrhythmia (Batchinsky et al., 2007; Draghici and Taylor, 2016; Duprey et al., 2019; Hayano and Yuda, 2021; Scavone et al., 2021). These bands are utilized to evaluate the balance between sympathetic and parasympathetic nervous system activity. By linking HRV to brain function and pathology, monitoring these signals may provide insights into the effects of treatments on cardiovascular function, enhancing understanding of brain-heart interactions and holding promise for improving diagnosis, prognosis, and therapeutic strategies.

Given the clinical relevance of HRV as a biomarker for various brain disorders, accurately extracting ECG from raw MEG recordings, and subsequently deriving HRV, can provide valuable physiological insights. This approach is especially advantageous for analyzing retrospective MEG datasets lacking simultaneous ECG recordings; it also simplifies subject preparation, minimizes artifacts from electrode placement (Godwin et al., 2024), and allows direct, time-locked assessment of brain-heart interactions without requiring separate ECG measurements.

Previous work has shown that ICA can isolate cardiac components from MEG data (Godwin et al., 2024), but this process often requires manual review and curation when the ICA output is noisy. Such manual intervention hampers reproducibility and scalability. Moreover, these methods do not include an automated RR interval outlier correction and rely exclusively on ICA for cardiac component extraction, without integrating complementary approaches.

In contrast, we present a novel algorithm for detecting R-peaks and computing HRV from MEG-derived cardiac components that reduces manual intervention to a single, transparent parameter: a user-set threshold. The algorithm can operate on cardiac signals obtained either from ICA or from MEG reference channels, providing flexibility when ICA separation is noisy or unavailable. In addition, processing reference channels offers a faster and computationally lighter alternative to ICA-based extraction (Supplementary Table 5).

The method employs a two-stage threshold-based process that allows the user to adjust detection sensitivity according to signal characteristics. In the first stage, the threshold can be tuned to automatically detect R-peaks with varying amplitudes in noisy signals, reducing the likelihood of missed detections. In the second stage, the algorithm automatically identifies aberrant RR intervals, often resulting from missed or spurious peaks, and corrects them using an adjustable threshold, rather than removing them. This design enhances robustness and transparency in HRV estimation, reduces processing time, avoids trimming data points, and maintains user control over signal quality optimization.

To assess performance, we compare the MEG-derived ECG signals and corresponding HRV metrics against simultaneously recorded independent ECG using time-domain, frequency-domain, and time–frequency signal processing techniques. Statistical analyses are applied to quantify signal similarity and agreement. The results demonstrate that raw MEG data can serve as a reliable source for ECG and HRV extraction, offering a semi-automated, flexible, and reproducible workflow that preserves all physiological information in the RR interval signal.

This approach differs from previous methods by combining semi-automation, flexibility in signal source, and robust RR interval correction, providing an efficient and reproducible framework for MEG-derived HRV analysis.

## 2 Methods

### 2.1 Data

This study used Brainstorm's MEG auditory dataset (Tadel et al., 2011; Bock et al., 2013), with prior consent from Brainstorm for research use of this publicly available dataset. The dataset comprises data from a single participant (ID: S01_AEF) recorded using the CTF 275-channel MEG system equipped with 26 reference sensors and 274 MEG axial gradiometers (one channel was disabled). Two acquisition runs of 6 min each were conducted with a 2,400 Hz sampling rate and an anti-aliasing low-pass filter set at 600 Hz. The data files were saved with the third-order gradient correction applied offline. During the acquisitions, the participant was stimulated binaurally using intra-aural earphones equipped with air tubes plus transducers. Simultaneously, the participant's ECG data were recorded using a separate bipolar channel.

Additionally, the Open MEG Archive resting-state sample dataset (Niso et al., 2016, 2024) was used in this study, which includes recordings from five participants (ID: sub-0002, sub-0003, sub-0004, sub-0006, and sub-0007) using a CTF 275-channel MEG system with 26 reference sensors and 270 MEG axial gradiometers. One acquisition run of 5 min length was conducted with a 2,400 Hz sampling rate and an anti-aliasing low-pass filter set at 600 Hz. The data files were saved with or without third-order gradient compensation. During the acquisition, participants rested with their eyes open, and ECG data were recorded using a separate bipolar channel.

The study received approval from the Atrium Health Wake Forest Baptist Institutional Review Board (IRB00110720). These datasets enabled an assessment of the accuracy of the extracted ECG and HRV from the MEG data in comparison to the simultaneously recorded ECG signal.

### 2.2 Data preprocessing

MNE-Python, an open-source Python package tailored for exploring, visualizing, and analyzing various types of human neurophysiological data, was utilized to preprocess the raw MEG data (Gramfort et al., 2013; Larson et al., 2024). Other libraries used extensively in all stages of data analysis include Matplotlib, NumPy, and SciPy (Hunter, 2007; Harris et al., 2020).

**Figure 1:**
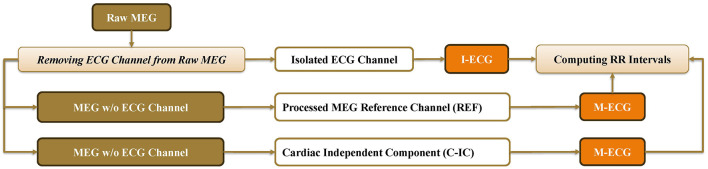
The preprocessing pipeline used in this study to isolate MEG-derived electrocardiogram (M-ECG) signals, including cardiac independent component (C-IC), independent electrocardiogram (I-ECG), and reference channel (REF).

The raw MEG data were resampled to 600 Hz to ensure a consistent sampling rate across all datasets (Supplementary Table 1). During preprocessing (Figure 1), the recorded ECG signal was extracted and saved as a separate channel. To ensure clarity, the separately recorded ECG signal is referred to as the independent ECG (I-ECG), and the ECG signal extracted from the MEG data as MEG-derived electrocardiogram (M-ECG). While the M-ECG signal originates from the raw MEG signals, it should not be confused with magnetocardiography, a distinct technique used to measure the magnetic fields produced by the electrical activity of the heart. Magnetocardiography specifically focuses on cardiac activity to assess heart function, detect abnormalities, and study cardiac disorders (Fenici et al., 2005).

The raw MEG data were bandpass filtered from 0.5 to 45 Hz (Wasimuddin et al., 2020) using a zero-phase FIR filter with a Hamming window, with the filter length automatically determined by MNE-Python to retain relevant ECG frequency information (Supplementary Table 1). A total of 27 channels from the right and left temporal regions (MRT/MLT 31–58), exhibiting a strong cardiac signature, were selected. ICA was performed using the FastICA algorithm from the MNE-Python library with a fixed random seed to ensure reproducibility. Through an iterative process, the ICA components of 15 proved sufficient in detecting the cardiac component (Supplementary Table 1). The component showing the most robust cardiac activity was then designated as a virtual channel, referred to as the cardiac independent component (C-IC; Figure 1). Similarly, the reference channel displaying the most robust cardiac activity was selected. To ensure clarity, the selected reference channel is referred to as REF. Together, C-IC and REF constitute the M-ECG channels (Figure 1).

The selection of C-IC and REF was performed by visual inspection of waveform morphology, focusing on the presence of a clear QRS-like pattern characteristic of cardiac activity. No automated selection metric, including correlation with I-ECG, spectral peak near heart rate, kurtosis, or template matching, was applied during this selection process. This selection was performed independently of any simultaneously recorded ECG and prior to HRV computation, ensuring that the process is not biased by the results, and that it remains fully applicable in MEG datasets where no ECG channel is available. While this procedure was not pre-registered, the validity of the visually identified components was confirmed *post hoc* by comparison with the I-ECG channel.

All three channels, I-ECG, C-IC, and REF, were baseline-corrected and normalized. C-IC and REF underwent discrete wavelet transform, using the PyWavelets library (Lee et al., 2019), employing a symlet-4 wavelet with a 3-level decomposition (Supplementary Table 1). From this decomposition, the deepest-level approximation coefficients (A3) were retained, corresponding to the broad, low-frequency component of the signal, while all detail coefficients (D1–D3) were set to zero prior to reconstruction. This choice emphasizes the cardiac-related low-frequency structure and maximizes QRS-related energy while attenuating high-frequency noise and muscle artifacts.

Signal quality was evaluated by assessing the temporal alignment of the detected R-peaks between M-ECG and I-ECG using a sequential peak-pairing algorithm, rather than conventional quantitative noise metrics such as peak-to-peak field variance, line noise signal-to-noise ratio, or cardio-component signal-to-noise ratio. This approach was chosen because, in these recordings, conventional metrics could indicate acceptable signal quality even when multiple R-peaks were misdetected, which would lead to unreliable HRV computation. Each M-ECG R-peak was paired to the nearest I-ECG R-peak in sequence, and the offset in seconds was computed. Peaks with offsets exceeding 0.02 s were considered misaligned. For each participant, the number and percentage of misaligned peaks were quantified, along with the mean and standard deviation of offsets. Additionally, a beat count agreement metric was calculated to provide a measure of overall correspondence between M-ECG and I-ECG beat counts. Recordings in which ≥3% of R-peaks were misaligned or in which the beat count agreement was below 98% were considered to have insufficient cardiac signal quality for reliable HRV computation (Supplementary Table 2). Of the six available recordings (one Brainstorm, five Open MEG Archive), three Open MEG Archive participants did not meet quality criteria and were excluded, leaving three participants (one Brainstorm, two Open MEG Archive) for analysis, ensuring that only high-quality cardiac data contributed to the HRV analyses (Supplementary Figure 1).

### 2.3 Computing RR intervals

To automatically detect valid R-peaks in the I-ECG and M-ECG signals, a two-stage, threshold-based algorithm was implemented, designed to robustly identify R-peaks and compute RR intervals, even in the presence of noise or artifacts. First, candidate peaks were identified using the SciPy signal processing library, which detects local maxima in a one-dimensional array. A minimum inter-peak distance of 0.5 s was applied to ensure physiologically plausible detections and prevent multiple peaks within a single cardiac cycle. The amplitudes of these candidate peaks were then analyzed to remove spurious detections. Specifically, for all candidate peaks with amplitudes *A*_*i*_ (where *i* indexes each detected peak and *N* is the total number of candidate peaks), the mean amplitude μ_*A*_, and standard deviation σ_*A*_ were computed as


$$\begin{array}{r} \mu_{A}=\frac{1}{N}\overset{N}{\underset{i=1}{\sum }}A_{i}\\ \sigma_{A}=\sqrt{\frac{1}{N}\overset{N}{\underset{i=1}{\sum }}{\left(A_{i}-\mu_{A}\right)}^{2}}.\; \end{array}$$

**Figure 2:**
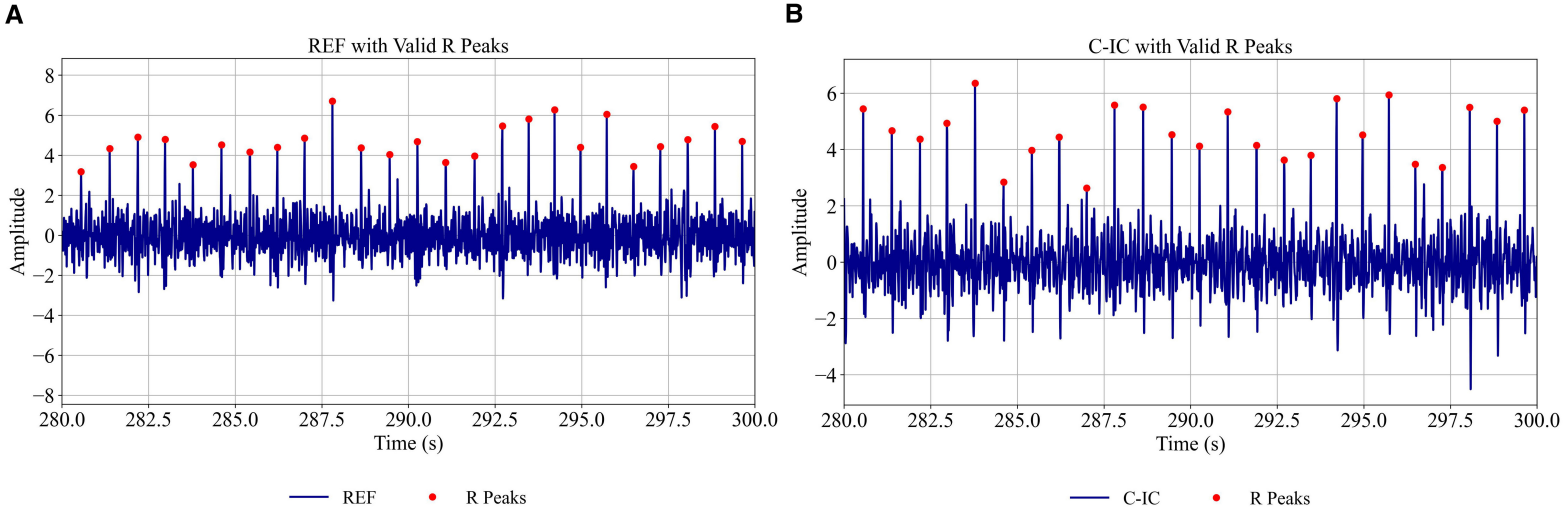
Automatically detected valid R-peaks for reference channel (REF) **(A)** and cardiac independent component (C-IC) **(B)** for participant 1 (280–300 s). Red dots indicate the detected valid peaks on each signal.

Peaks were retained only if their amplitudes satisfied the criterion:


$$\begin{array}{l} \mu_{A}-n\cdot \sigma_{A}\leq A_{i}\leq \mu_{A}+n\cdot \sigma_{A}, \end{array}$$

where the default multiplier (*n*) was 2 for both the upper and lower thresholds (Supplementary Table 1). These thresholds are user-adjustable, allowing the algorithm to adapt to signals with varying quality and noise levels, ensuring that only consistent and reliable R-peaks are retained (Figure 2, Supplementary Figures 2, 3). Peaks outside this range were considered invalid and excluded from further analysis.

Following peak detection, RR intervals were computed as the time differences between consecutive valid R-peaks:


$$\begin{array}{l} RR_{i}=t_{i+1}-t_{i},\; \end{array}$$

where (*t*_*i*_) denotes the time of the (*i*)-th R-peak, and *RR*_*i*_ represents the duration of the cardiac cycle between peaks *i* and *i*+ 1.

Before performing outlier correction, each RR interval was paired with its cumulative temporal position, forming a two-dimensional array (*x*_*i*_, *RR*_*i*_), where *x*_*i*_ represents the cumulative time elapsed from the first detected peak up to the *i*-th interval:


$$\begin{array}{l} x_{i}=\overset{i}{\underset{k=\;1}{\sum }}RR_{k}. \end{array}$$

This design preserves the true temporal continuity of the cardiac signal: even when outlier intervals are later modified or set to zero, the cumulative time axis remains physiologically accurate.

To minimize the influence of missed or spurious peaks, these RR intervals were evaluated for outliers using a similar statistical approach. Intervals were classified as outliers if they fell outside the range defined by the mean (μ_*RR*_) plus or minus a user-set multiple (*n*) of the standard deviation (σ_*RR*_), with the default multiplier set to 1.5 for both the upper and lower thresholds (Supplementary Table 1):


$$\begin{array}{l} \mu_{RR}-n\cdot \sigma_{RR}\leq RR_{i}\leq \mu_{RR}+n\cdot \sigma_{RR}.\; \end{array}$$

**Figure 3:**
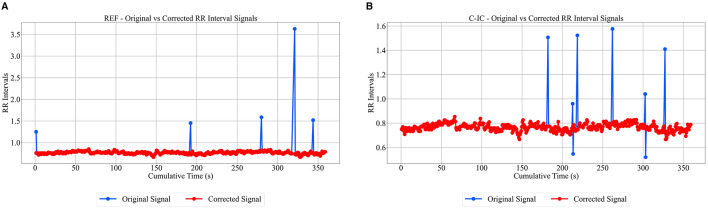
Original vs. corrected RR intervals for reference channel (REF) **(A)** and cardiac independent component (C-IC) **(B)** for participant 1 (0–360 s). The corrected signal is shown in red, while the uncorrected signal appears in blue.

Outlier intervals were temporarily set to zero to prevent distortion of the RR signal, and continuity was subsequently restored through linear interpolation, filling gaps with estimated values based on neighboring valid intervals. Because the cumulative time structure (*x*_*i*_) was defined before correction, interpolation preserves the physiological timing of cardiac cycles, maintaining consistency between the original and corrected RR series (Figure 3).

To directly benchmark M-ECG R-peak detection against I-ECG, per-subject performance metrics, including sensitivity, positive predictive value, and F1 score, were computed using a ±0.05 s tolerance window (Supplementary Table 3).

By combining amplitude-based R-peak detection with RR interval outlier correction and interpolation within a temporally consistent framework, this algorithm provides a robust, semi-automated method for HRV extraction from both M-ECG and I-ECG signals.

### 2.4 Time domain

**Figure 4:**
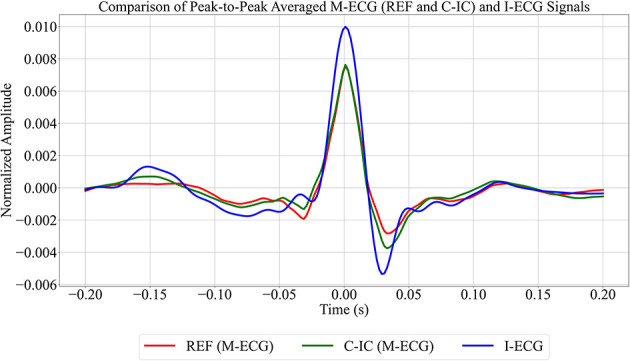
Comparison of the averaged peak-to-peak reference channel (REF), cardiac independent component (C-IC), and independent electrocardiogram (I-ECG) signals for participant 1. REF is shown in red, C-IC in green, and I-ECG in blue.

**Figure 5:**
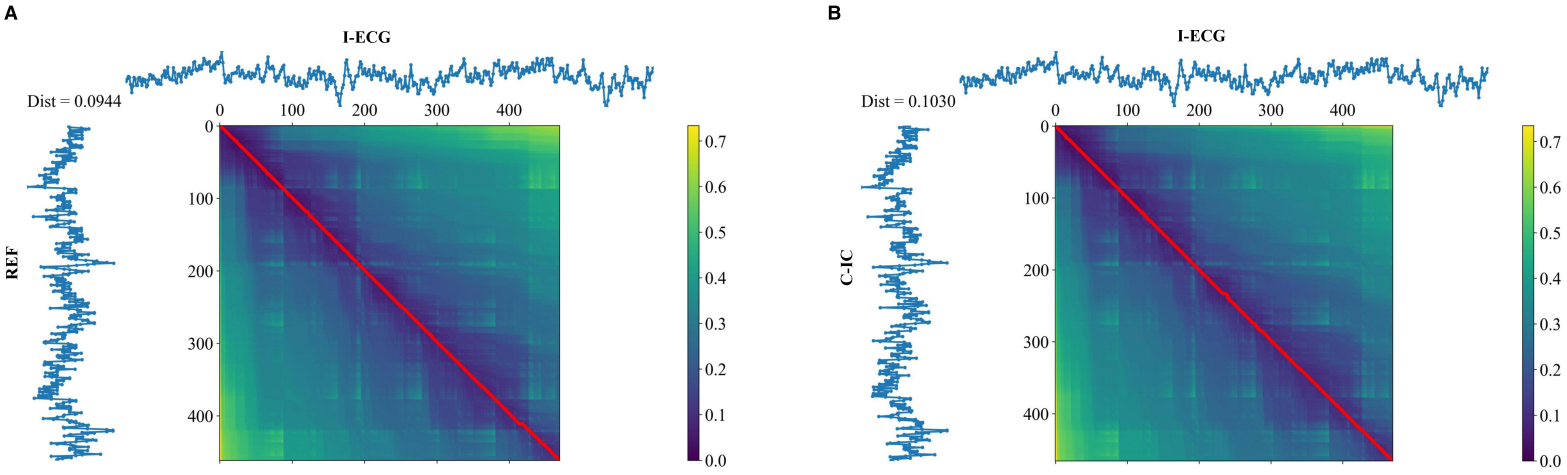
Dynamic time warping (DTW) of RR intervals (seconds) with independent electrocardiogram (I-ECG) vs. reference channel (REF) **(A)**, and I-ECG vs. cardiac independent component (C-IC) **(B)** for participant 1. The lower the DTW value, the more similar the two signals are, with a value closer to zero indicating a better alignment and higher similarity.

To allow for clearer visualization of consistent peak patterns between the M-ECG and I-ECG signals, peak-to-peak signal averaging around R-peaks was computed for these signals (Figure 4, Supplementary Figures 4, 5). Additionally, to compare time series sequences between I-ECG RR intervals and M-ECG RR intervals, dynamic time warping was computed (Figure 5, Supplementary Figures 6, 7) using the DTAIDistance Python library (Meert et al., 2020). Dynamic time warping is a powerful technique that allows for comparing time series data that may vary in length or timing by finding an optimal alignment path between the sequences, enabling comparison and similarity measurement even when the sequences have different lengths or rates of change (Senin, 2008).

Widely-used time-domain HRV indices, including the standard deviation of normal-to-normal intervals, which reflects overall variability, and the root mean square of successive differences, which estimates vagally mediated changes in HRV (Munoz et al., 2015; Shaffer and Ginsberg, 2017), were computed for both I-ECG and M-ECG signals (Table 1). Comparing the standard deviation of normal-to-normal intervals and the root mean square of successive differences between the two signals allowed for assessing their similarity in capturing HRV.

**Table 1:** RR intervals comparison from MEG-derived electrocardiogram (M-ECG)—specifically cardiac independent component (C-IC) and reference channel (REF)—and independent electrocardiogram (I-ECG) signals over 0–200 s in three participants.

**Metrics**			**Participant 1**			**Participant 2**			**Participant 3**		
			**REF**	**I-ECG**	**C-IC**	**REF**	**I-ECG**	**C-IC**	**REF**	**I-ECG**	**C-IC**
SDNN (s)			0.03	0.03	0.03	0.03	0.03	0.03	0.11	0.11	0.11
RMSSD (s)			0.02	0.02	0.02	0.02	0.02	0.02	0.12	0.1	0.1
SD (s)			0.03	0.03	0.03	0.03	0.03	0.03	0.11	0.11	0.11
Mean RR (s)			0.77	0.77	0.77	0.73	0.73	0.73	0.86	0.87	0.87
CV			0.04	0.04	0.04	0.04	0.04	0.04	0.13	0.13	0.13
95% CI (s)		Upper bound	0.82	0.82	0.82	0.80	0.81	0.81	1.08	1.08	1.08
		Lower bound	0.71	0.71	0.71	0.67	0.67	0.67	0.68	0.68	0.68
REF vs. I-ECG	Spearman's correlation		0.73			0.99			0.97		
	RMSE (s)		0.02			0.01			0.03		
	MAE (s)		0.02			0			0		
	Bland–Altman (s)	Upper LoA	0.0160			0.0229			0.0859		
		Mean difference	0.0003			−0.0011			−0.0030		
		Lower LoA	−0.0154			−0.0252			−0.0919		
	Mann–Whitney *U*-test	Statistic	33773			33526			33593		
		*P*-value	0.988			0.873			0.904		
	Mann–Whitney *U*-test (PSD)	Statistic	971			967			1000		
		*P*-value	0.983			0.997			0.793		
C-IC vs. I-ECG	Spearman's correlation		1			1			1		
	RMSE (s)		0.02			0			0		
	MAE (s)		0.01			0			0		
	Bland–Altman (s)	Upper LoA	0.0102			0.0013			0.0021		
		Mean difference	0.0002			0			0		
		Lower LoA	−0.0097			−0.0013			−0.0021		
	Mann–Whitney *U*-test	Statistic	33858.5			33800			33809		
		*P*-value	0.973			1.00			0.996		
	Mann–Whitney *U*-test (PSD)	Statistic	997			973			968		
		*P*-value	0.812			0.970			1.000		

All HRV and error metrics are reported in seconds. 95% confidence intervals (CI) refer to RR intervals (seconds).

SDNN, standard deviation of normal-to-normal intervals; RMSSD, root mean square of successive differences; SD, standard deviation; CV, coefficient of variation; RMSE, root mean square error; MAE, mean absolute error; PSD, power spectral density.

### 2.5 Frequency and time-frequency domains

**Figure 6:**
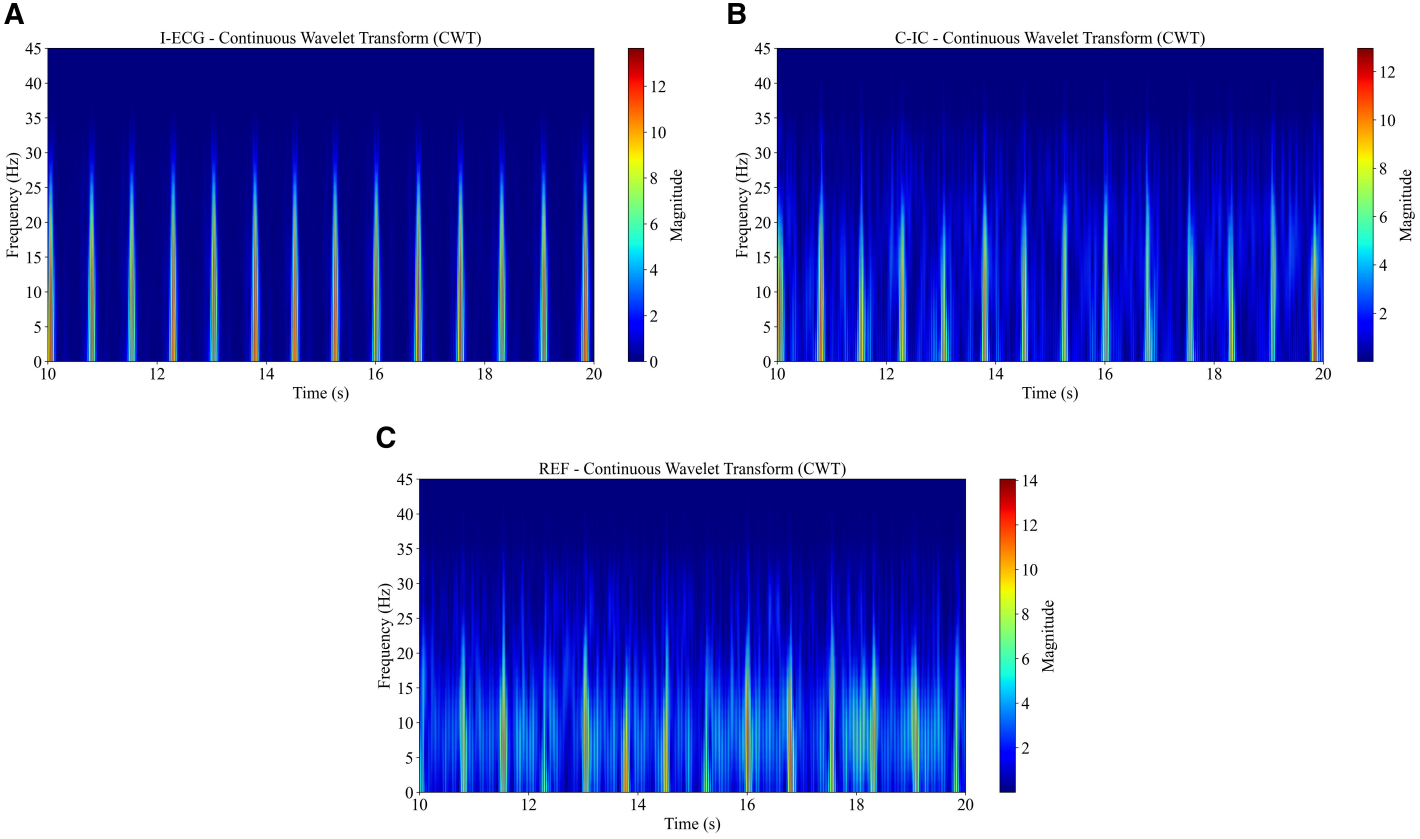
The continuous wavelet transform (CWT) analysis using the Morlet wavelet reveals time–frequency patterns in independent electrocardiogram (I-ECG) **(A)**, cardiac independent component (C-IC) **(B)**, and reference channel (REF) **(C)** signals from participant 1, with recurrent high-power bursts over 20 s corresponding to R waves.

To investigate transient events, frequency modulation, and other dynamic characteristics of I-ECG and M-ECG signals, continuous wavelet transform was employed due to its high time-frequency resolution. This method, implemented using the Scipy and PyWavelet libraries (Lee et al., 2019; Virtanen et al., 2020), allowed for the examination of signals with localized features that change in frequency over time. To visualize the results of these analyses, spectrograms of time and frequency were generated (Figure 6).

Since frequency and time-frequency analyses often assume evenly sampled data, the uneven sampling of RR intervals, due to natural HRV, posed a potential limitation (Hashimoto et al., 2013). To mitigate this, cubic spline interpolation was used to convert the unevenly sampled RR intervals into evenly sampled signals, facilitating accurate frequency and time-frequency analyses. Cubic spline interpolation achieves this by fitting a piecewise cubic polynomial between adjacent data points (de Boor, 2001; Dyer and Dyer, 2001), ensuring smoothness and continuity in the interpolated signal. The fixed interpolation length of 850 points was chosen to provide sufficient temporal resolution to capture RR interval variability across the recording while maintaining smoothness in the cubic spline interpolation, resulting in a uniform sampling rate of approximately 2.37 Hz (Supplementary Table 1).

**Figure 7:**
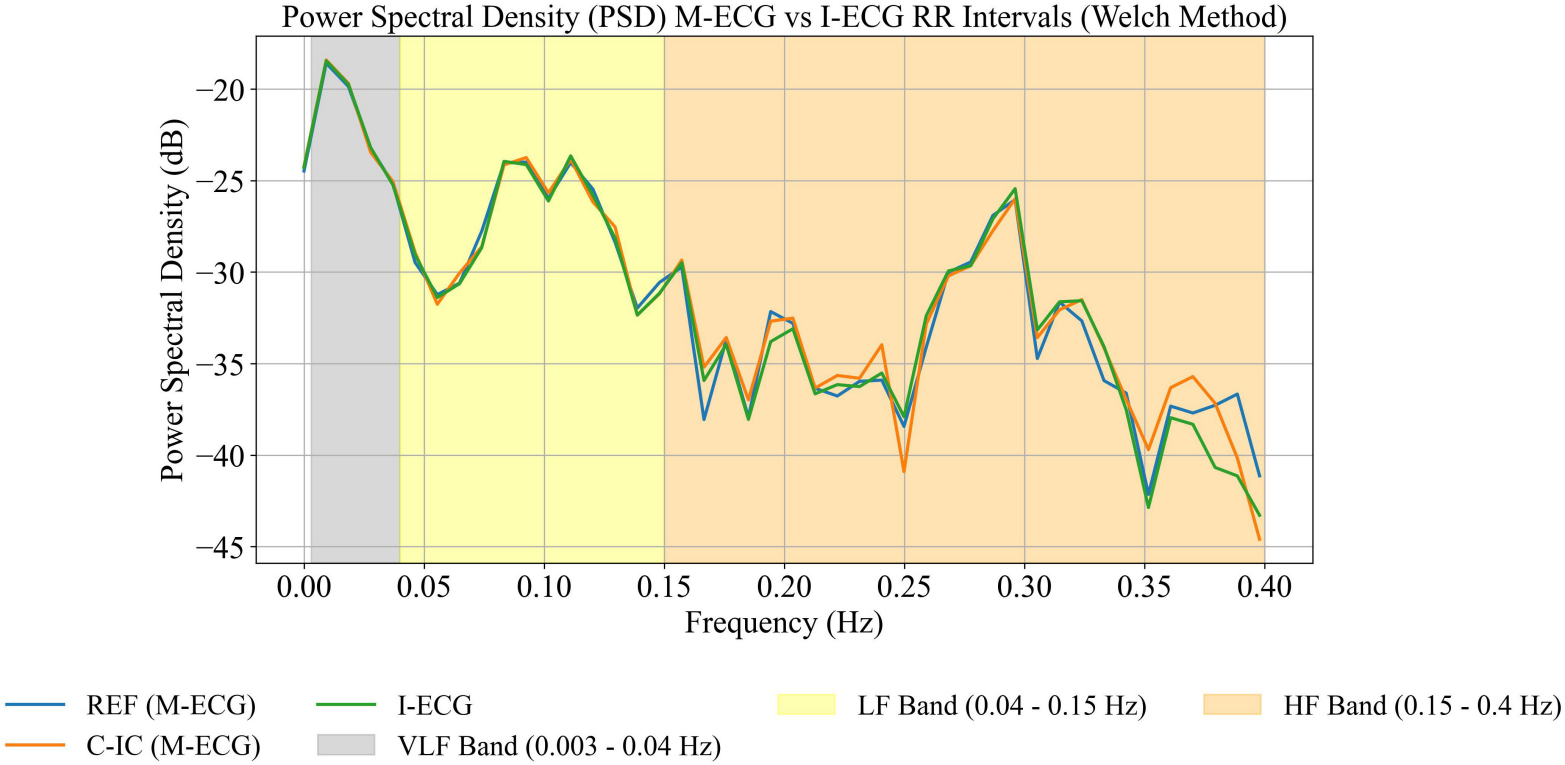
Power spectral density (PSD) between reference channel (REF), cardiac independent component (C-IC), and independent electrocardiogram (I-ECG) RR intervals for participant 1 (0–360 s). Low frequency (LF) and high frequency (HF) bands are analyzed across REF (blue), C-IC (orange), and I-ECG (green) signals. Very-low-frequency (VLF) band is shown for completeness but is not interpreted due to short recording duration.

To assess the frequency-domain properties and interrelations of the I-ECG and M-ECG interpolated RR intervals, power spectral density analysis was performed using Welch's method on the interpolated signals (Figure 7, Supplementary Figures 8, 9). Default MNE-Python parameters were used unless specified: Hamming window, segment length of 256, constant detrending, and no segment overlap (Supplementary Table 1).

### 2.6 Statistical analyses

To measure the similarity between I-ECG and M-ECG RR intervals, several statistical metrics were computed. Standard deviation was used to assess the variability and precision of RR interval measurements derived from M-ECG and I-ECG. To quantify the average difference between I-ECG and M-ECG RR intervals, root mean squared error and mean absolute error were calculated, while the coefficient of variation was calculated to measure the relative variability of both sets of intervals. 95% confidence intervals were computed for the RR intervals themselves (in seconds) using the 2.5th and 97.5th percentiles, providing an estimate of the range within which the RR interval values are likely to fall and reflecting the precision of the measurements. The Spearman rank correlation coefficient was also computed to gauge the correlation strength of M-ECG and I-ECG RR intervals (Table 1).

To determine if RR intervals from I-ECG and M-ECG were drawn from populations with the same distribution, the Mann–Whitney *U*-test was performed. This test assessed whether there were significant differences (temporally and frequency-wise) in the distributions of RR intervals between I-ECG and M-ECG. Statistical analyses were performed using Scipy and Scikit-learn libraries (Pedregosa et al., 2011; Virtanen et al., 2020). Bland–Altman analysis (Martin Bland and Altman, 1986) was conducted to compare the mean differences between sets of RR intervals derived from M-ECG and I-ECG, identifying any systematic bias or proportional differences between the two measurement methods (Table 1; Supplementary Figures 10–12).

To assess paired agreement, Lin's concordance correlation coefficient was computed for mean RR and root mean square of successive differences between M-ECG and I-ECG (Supplementary Table 4). Lin's concordance correlation coefficient was selected because it evaluates both precision and accuracy in paired measurements and remains mathematically valid with small sample sizes. These HRV metrics were calculated in non-overlapping windows of 30 consecutive RR intervals to reduce the influence of short-term fluctuations. Standard deviation of normal-to-normal intervals was not analyzed in these short windows because its variability estimates are unreliable with only 30 RR intervals per window. Similarly, equivalence testing using two one-sided tests with clinician-specified bounds was not performed, as the limited number of RR interval windows would result in insufficient statistical power for meaningful inference. All computations were performed in Python, and the custom analytical scripts of this study are made available on GitHub (release v1.0.0, commit 1d5faec) at https://github.com/izadysadr/MEG-HRV-Extraction, which includes full documentation and the release history, to facilitate replication.

## 3 Results

### 3.1 Algorithm

The low/high thresholding approach effectively filtered out invalid peaks, retaining only consistent and physiologically plausible R-peaks while excluding artifacts (Figure 2, Supplementary Figures 2, 3). Per-subject benchmarking of M-ECG R-peaks against I-ECG using a ±0.05 s tolerance window showed high sensitivity, positive predictive value, and F1 scores (Supplementary Table 3), confirming the reliability of the detected RR intervals. Additionally, the algorithm identified and interpolated outliers in the RR intervals (Figure 3), ensuring a smooth and continuous time series. Together, these steps helped mitigate the impact of artifacts and outliers, thereby enhancing the reliability of the computed RR interval sequences.

### 3.2 Time domain

A comparison of peak-to-peak signal averaging between I-ECG and M-ECG revealed that while the peak amplitude of I-ECG is slightly higher than that of M-ECG, the alignment of the average peak amplitude between the signals indicates a match in capturing the R-peaks (Figure 4, Supplementary Figures 4, 5).

The dynamic time warping analysis revealed a distance of approximately 0.094 s between REF and I-ECG RR intervals and 0.103 s between C-IC and I-ECG RR intervals for participant 1 (Figure 5). Given that the average RR interval for participant 1 was approximately 0.77 s (corresponding to a mean heart rate of approximately 78 bpm), these dynamic time warping distances represent a small temporal deviation of approximately 12%−14% relative to the average cardiac cycle, indicating high fidelity in capturing heartbeat timing. The nearly straight alignment path observed in Figure 5 (participant 1) and Supplementary Figures 6, 7 (participants 2 and 3) further confirms a close temporal alignment between M-ECG and I-ECG RR intervals.

### 3.3 Frequency domain

The power spectral density analysis of REF, C-IC, and I-ECG across low-frequency and high-frequency bands (Figure 7, Supplementary Figures 8, 9) demonstrates that REF and C-IC RR intervals retain the spectral distributions observed in the I-ECG RR intervals. Very-low-frequency estimates are shown for completeness but are not interpreted, as short recording durations preclude reliable resolution of this band.

### 3.4 Statistics

The statistical comparison of the RR intervals derived from M-ECG (REF and C-IC) against those from I-ECG for all three participants (Table 1) highlights the similarity and strong agreement between the two sets of signals. The standard deviation of NN intervals, root mean square of successive differences, standard deviation, mean RR, coefficient of variation, and confidence interval values for both REF and C-IC are nearly identical to those for I-ECG in each participant. Both REF and C-IC exhibit high Spearman's rank correlation values with I-ECG across participants. In addition, root mean square error and mean absolute error values appear close to zero in each participant, confirming the similarity between the M-ECG and I-ECG RR intervals.

Lin's concordance correlation coefficient further quantified paired agreement between M-ECG and I-ECG (Supplementary Table 4). Both REF and C-IC demonstrated strong concordance with I-ECG for mean RR intervals, reflecting accurate capture of overall cardiac cycle timing. RMSSD values also showed high agreement, indicating that M-ECG channels reliably represented short-term variability in RR intervals.

The Bland–Altman analysis revealed negligible mean bias and tight limits of agreement between M-ECG and I-ECG for each participant, suggesting high agreement between the signals (Table 1; Supplementary Figures 10–12). Furthermore, the Mann–Whitney *U*-tests yielded no significant statistical differences between the signal sets in each participant.

## 4 Discussion

This study aimed to evaluate the efficacy of M-ECG in capturing cardiac activity and to compare these signals to the I-ECG recording. Using a rigorous preprocessing and analysis pipeline, multiple aspects of the signals, including RR interval computation, time-domain indices, frequency-domain characteristics, and statistical testing, were examined. The preprocessing pipeline employed for extracting the cardiac signals from MEG data was effective in isolating cardiac components. The study successfully identified and designated the cardiac signals using REF and C-IC as M-ECG channels. The use of bandpass filtering of 0.5–45 Hz ensured that only the most relevant cardiac-related features were retained.

The tailored algorithm for detecting R-peaks demonstrated high precision in excluding invalid peaks and minimizing artifacts. The thresholding approach ensured that physiologically plausible peaks were retained (Supplementary Table 3, Figure 2, Supplementary Figure 2, 3), while outlier removal and linear interpolation provided a smooth and continuous RR interval series (Figure 3). These steps allowed for maintaining the integrity of HRV analyses, particularly when working with noisy or artifact-prone data.

Our results demonstrate that cardiac activity can be reliably recovered from standard MEG recordings and used to compute HRV metrics that closely match those from a simultaneously recorded ECG. Specifically, the RR interval time series derived from both C-IC and REF channels showed nearly identical statistical properties to I-ECG. Time-domain HRV indices such as standard deviation of NN intervals and root mean square of successive differences were virtually indistinguishable between M-ECG and I-ECG, and Spearman correlations of the RR-interval series were remarkably high (Table 1). Lin's concordance correlation coefficient further confirmed strong paired agreement between M-ECG and I-ECG, with both channels accurately capturing mean RR intervals and short-term variability as reflected by the root mean square of successive differences (Supplementary Table 4). Bland–Altman analysis revealed negligible mean bias and tight limits of agreement between M-ECG and I-ECG intervals (Table 1; Supplementary Figures 10–12). The spectral (frequency-domain) profiles of the RR-interval signals, across low-frequency and high-frequency bands, were likewise closely aligned (Figure 7, Supplementary Figures 8, 9), while very-low-frequency estimates are shown for completeness but are not interpreted due to the short recording duration. These findings indicate that our algorithm successfully extracts the cardiac signal from MEG data such that the derived HRV metrics are virtually interchangeable with those from a true ECG.

This outcome aligns with recent work that developed an automated artificial intelligence-based ICA pipeline to identify the cardiac component in MEG data, reporting strong concordance between MEG-derived and ECG-derived HRV metrics (Godwin et al., 2024). However, unlike these ICA-only approaches, which typically require manual inspection, lack automated RR interval correction, and solely rely on ICA to identify cardiac components, our method offers a semi-automated and flexible alternative. It can operate on cardiac signals derived either from ICA or from MEG reference channels, providing a faster and computationally lighter option (Supplementary Table 5), especially when ICA separation is noisy or unavailable. Because using reference channels bypasses ICA decomposition, it also scales more efficiently for batch processing of large archival datasets, whereas ICA can become the computational bottleneck in high-throughput workflows. To minimize manual intervention, the algorithm employs a two-stage threshold-based process: the first stage detects R-peaks with varying amplitudes in noisy signals, while the second stage identifies and corrects aberrant RR intervals without discarding data points. This design enhances robustness, transparency, and reproducibility in HRV estimation, reduces manual workload and processing time, and maintains user control over signal quality. By preserving all physiological information in the RR interval series, this workflow provides an efficient and practical framework for MEG-derived HRV analysis.

Recovering HRV from MEG data has several important implications. It enriches the information obtained in any MEG study by adding a cardiovascular dimension to the neural data. Traditionally, MEG analysis treats cardiac fields as noise to be removed (Escudero et al., 2007; Breuer et al., 2014); we show that these fields are in fact a valuable signal. This means that many existing MEG datasets (even archival ones) could retrospectively be mined for autonomic measures without requiring separate ECG channels. Such a multimodal perspective aligns with a growing interest in brain-heart interactions. For example, stroke, dementia, traumatic brain injury, post-traumatic stress disorder, and sudden unexpected death in epilepsy carry elevated cardiovascular risk (Samuels, 2007; Tan et al., 2011; Baysal-Kirac et al., 2017; Deckers et al., 2017; Wolters et al., 2018; Schnabel et al., 2021; Song et al., 2021; Stewart et al., 2022). By extracting M-ECG, future studies can simultaneously monitor neural oscillations and autonomic state, potentially yielding new insights into disease mechanisms and brain-body coupling.

Moreover, this capability could streamline clinical MEG protocols. Since M-ECG already registers the cardiac waveform (as our C-IC and REF channels show), hospitals conducting MEG for epilepsy or research could compute HRV without extra electrodes. M-ECG opens the door to truly multimodal neuro-cardiac imaging, bringing cardiovascular biomarkers into the fold of neuroimaging. The M-ECG extraction method can be adapted for use with various MEG systems, allowing for the extraction of normal and abnormal HRV, which can serve as a valuable biomarker for neurological disorders closely linked to autonomic nervous system dysregulation. Extracting M-ECG facilitates the retrospective analysis of the relationship between HRV and neuronal function in patients with cardiac and neurological conditions, providing deeper insights into disease mechanisms and advancing both diagnostic and therapeutic strategies.

This work paves the way for future studies. We plan to validate the algorithm in larger and more varied cohorts, including older adults and patients with autonomic or cardiac disorders. It will be important to test whether M-ECG remains accurate in the presence of arrhythmias, tachycardia, or other ECG anomalies. Additionally, pulsatile blood flow and pulse rate variability may contribute to the M-ECG signals; while our algorithm primarily targets R-peak-derived HRV, some components of the M-ECG signal may reflect pulse rate variability, and future studies should consider their potential impact on HRV extraction. We also intend to apply the method in different MEG protocols, namely longer recording sessions and both task-based and resting-state conditions.

Our study included three healthy participants, consistent with the goals of an initial feasibility investigation. Although the sample size is limited, it provides a preliminary demonstration of the approach. As is typical in early-stage research, future studies in more diverse populations will be important for broader validation. Our algorithm's performance may be influenced by factors such as low-amplitude cardiac artifacts or elevated noise levels. The recording duration (5–6 min) was sufficient for initial HRV analysis, though longer recordings may offer additional insights into long-term variability. Because of the relatively short recordings, spectral estimates in the very-low-frequency band are inherently unreliable; while very-low-frequency results are presented for completeness, they are not interpreted (Figure 7). These limitations suggest that our findings reflect proof-of-concept performance rather than a clinically validated solution.

## 5 Conclusions

In conclusion, this study introduces a novel algorithm for extracting cardiac activity from MEG data to accurately approximate HRV measures from actual ECG recordings. Through analysis across various domains, including time, frequency, and time-frequency, as well as statistical assessments, the robustness and comparability of both C-IC and REF in capturing HRV and cardiac dynamics were demonstrated. The findings suggest the potential clinical utility of M-ECG in exploring brain-heart interactions and advancing neurocardiac research.

## Data Availability

The data analyzed in this study were obtained from Brainstorm's MEG auditory dataset (permission required) and the Open MEG Archive resting-state sample dataset (publicly available). The derived data and analyses are included in the article and/or Supplementary material. The analysis code is publicly available on GitHub (https://github.com/izadysadr/MEG-HRV-Extraction). Further inquiries can be directed to the corresponding authors.
