# Supplementary material for: M-ECG: extracting heart signals with a novel computational analysis of magnetoencephalography data
Source: Front Neuroimaging. 2026 Jan 9;4:1675960. doi: 10.3389/fnimg.2025.1675960 (PMC12827190; doi:10.3389/fnimg.2025.1675960)
Supplement: Supplementary file 1 [file Data_Sheet_1.docx]

**Supplemental Figures and Tables**


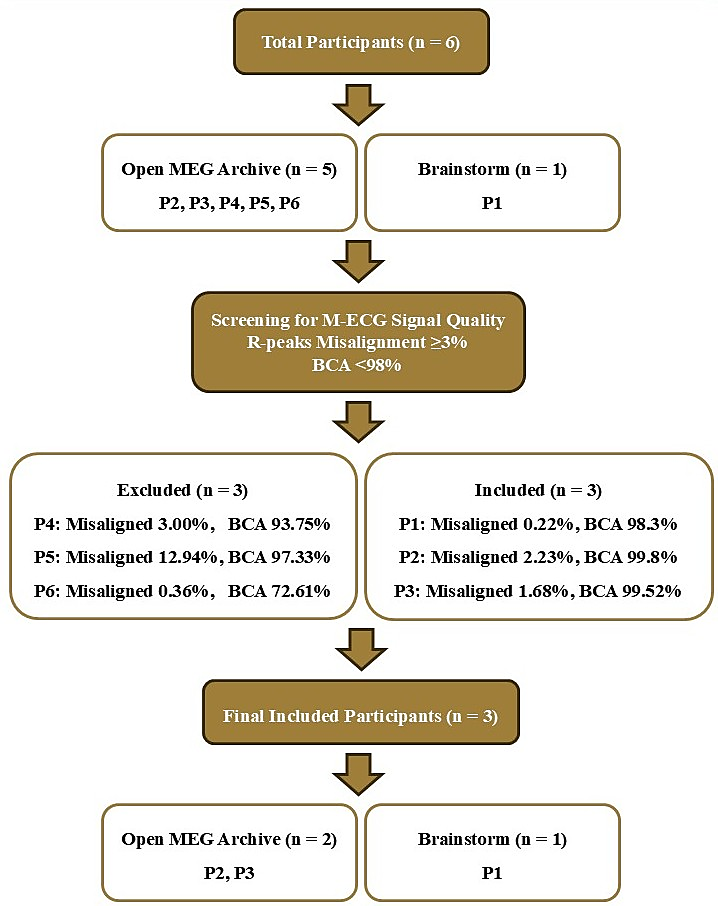


**Supplemental Figure 1.** Participant selection flow depicting screening, exclusions, and final inclusion based on signal quality.


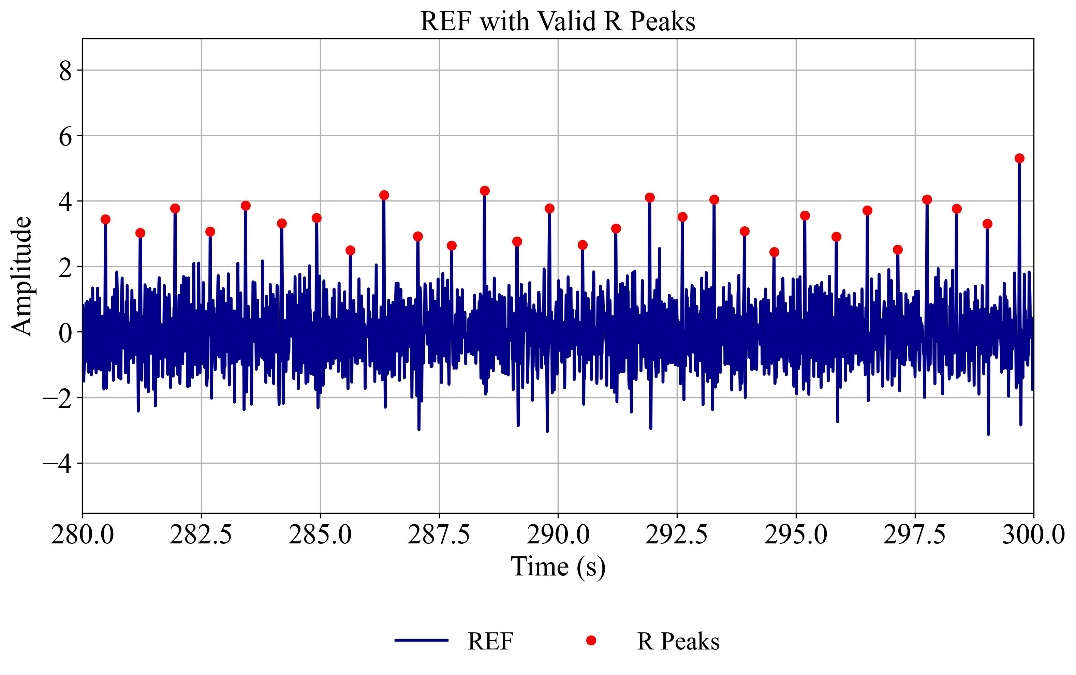


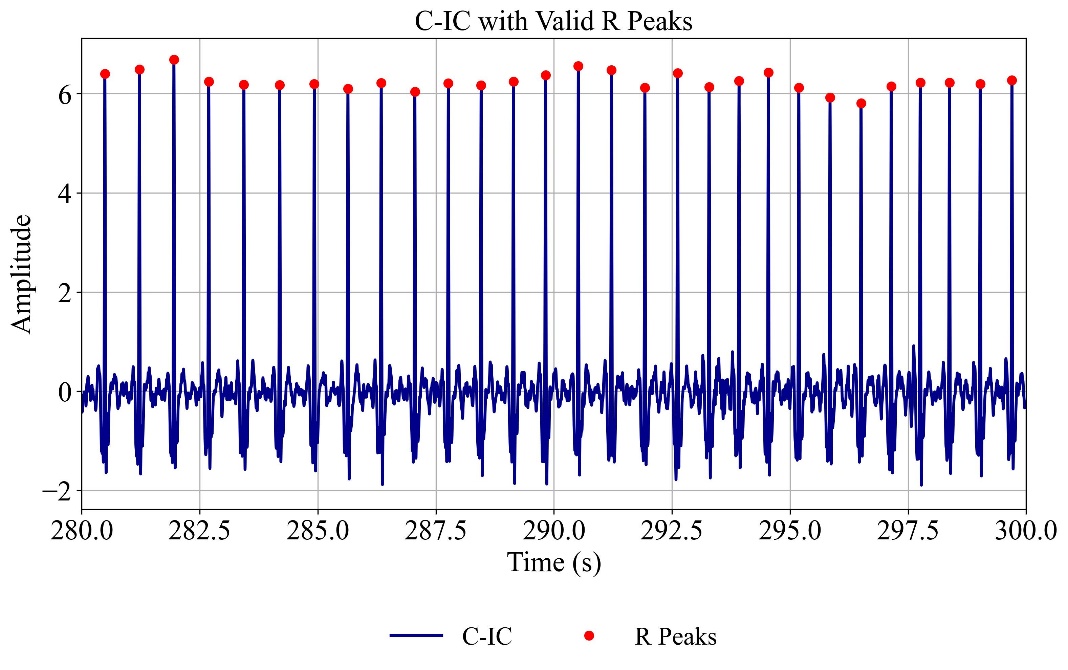


**Supplemental Figure 2.** Automatically detected valid R peaks for reference channel (REF) and cardiac independent component (C-IC) (280 – 300 s, participant 2).


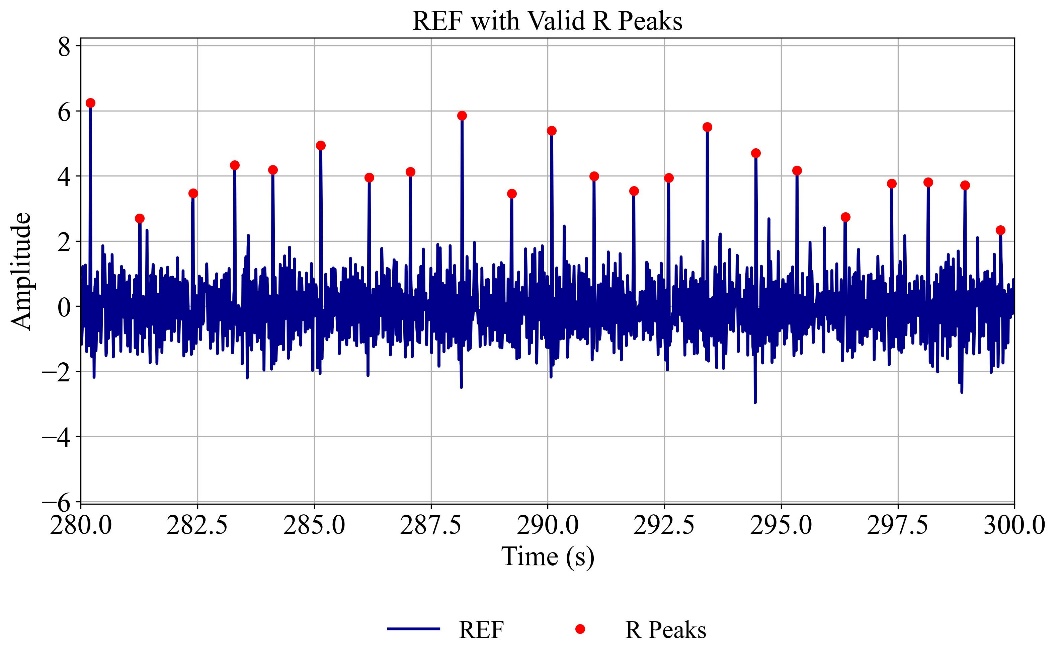


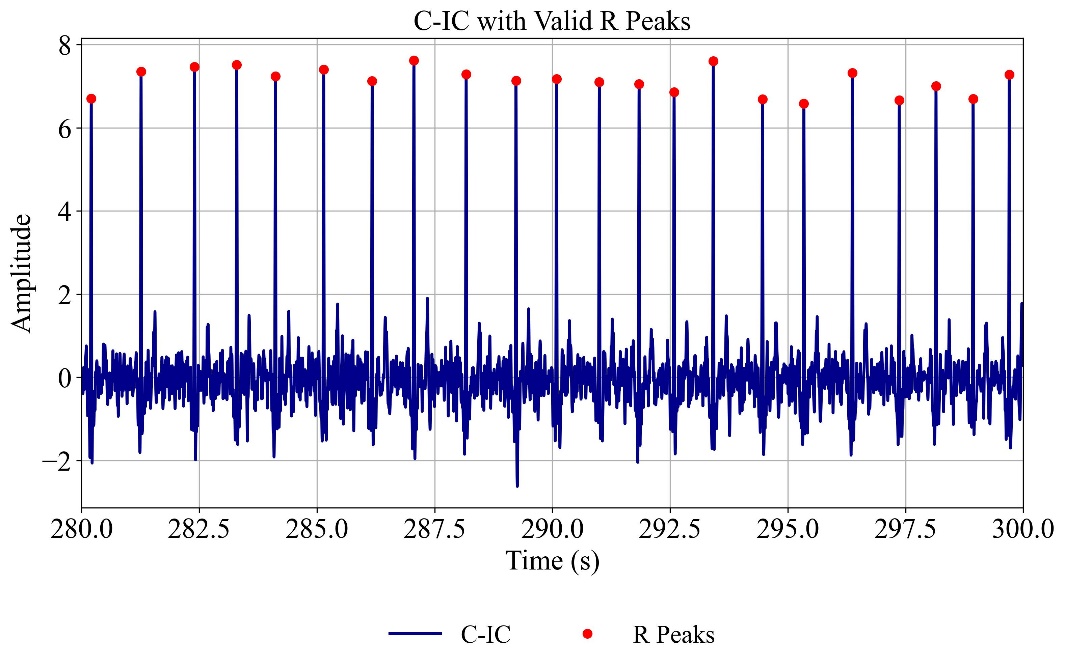


**Supplemental Figure 3.** Automatically detected valid R peaks for reference channel (REF) and cardiac independent component (C-IC) (280 – 300 s, participant 3).


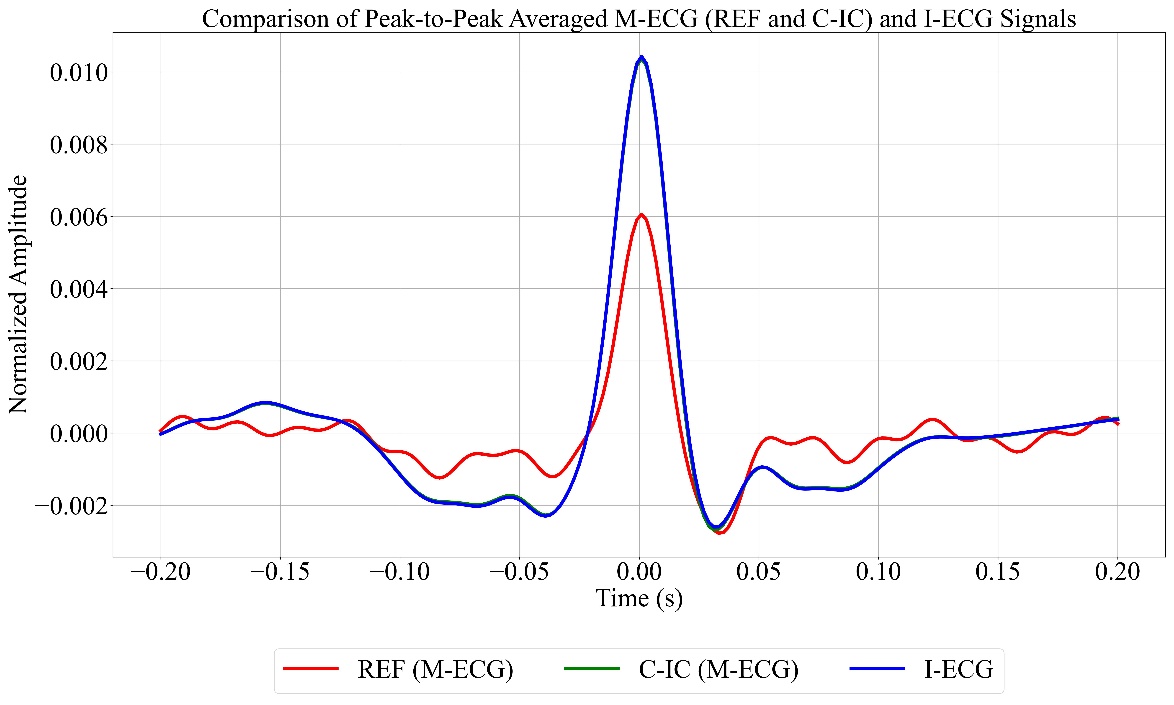


**Supplemental Figure 4.** Averaged peak-to-peak comparison of the reference channel (REF), cardiac independent component (C-IC), and independent electrocardiogram (I-ECG) signals with overlapping C-IC and I-ECG values (participant 2).


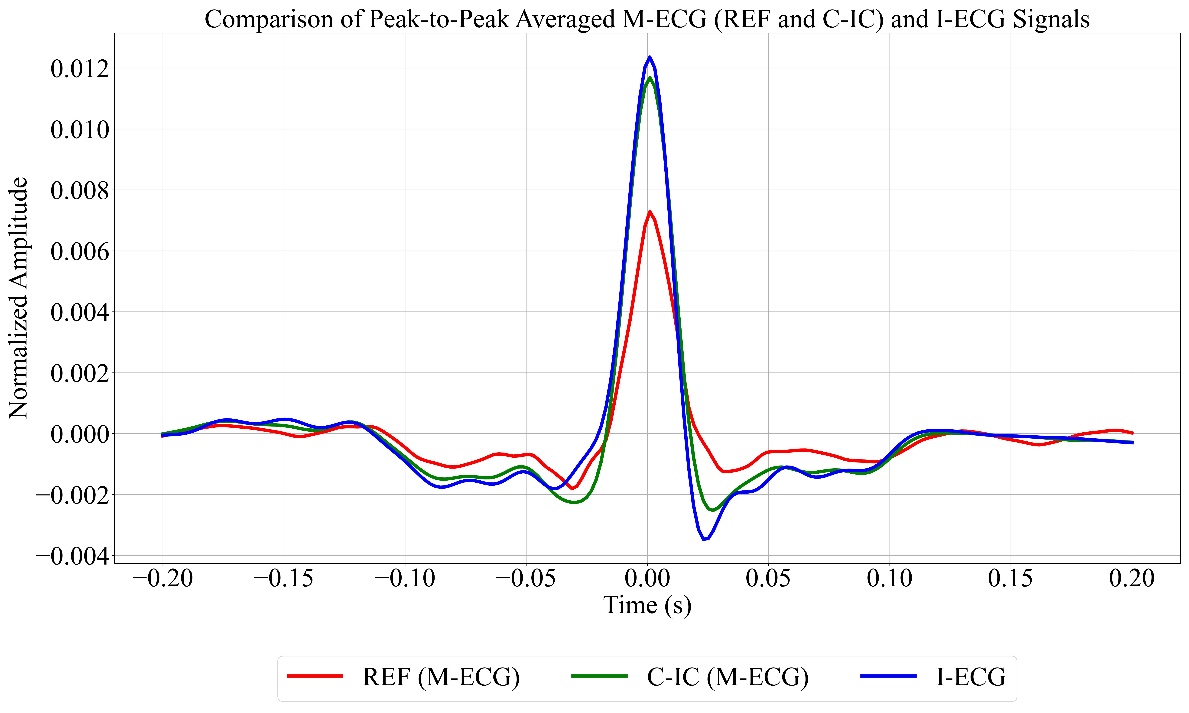


**Supplemental Figure 5.** Averaged peak-to-peak comparison of the reference channel (REF), cardiac independent component (C-IC), and independent electrocardiogram (I-ECG) signals (participant 3).

|  | **I-ECG** |
| --- | --- |
| **REF** | 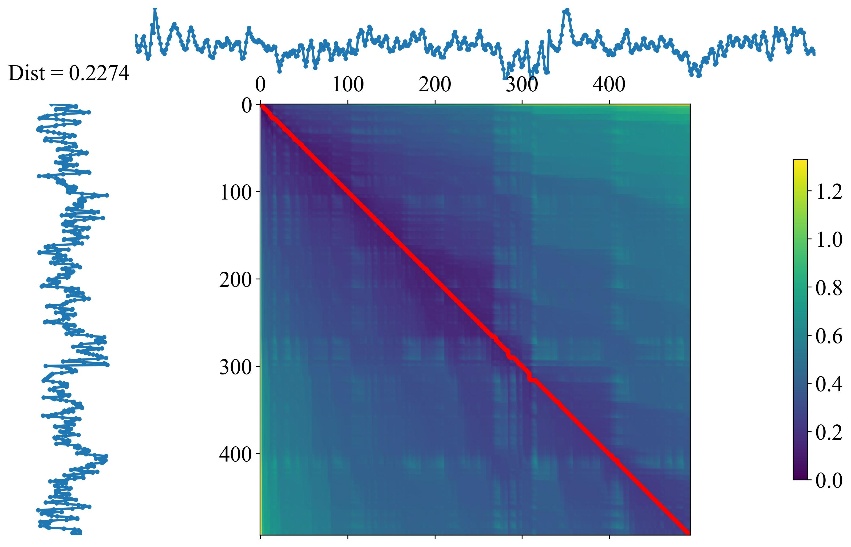 |
|  |  |
| **C-IC** | 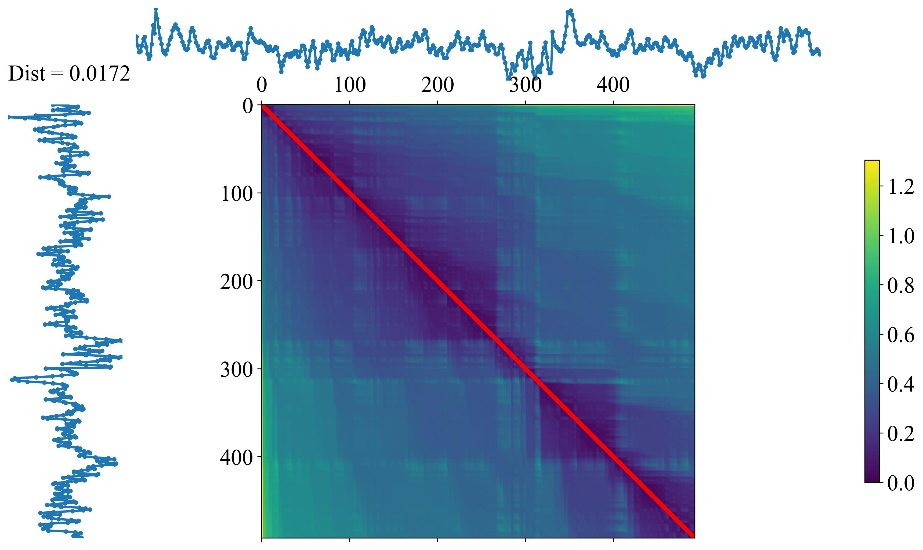 |

**Supplemental Figure 6.** Dynamic time warping (DTW) of RR intervals (seconds) with independent electrocardiogram (I-ECG) versus reference channel (REF) **(top)**, and I-ECG versus cardiac independent component (C-IC) **(bottom)** (participant 2).

|  | **I-ECG** |
| --- | --- |
| **REF** | 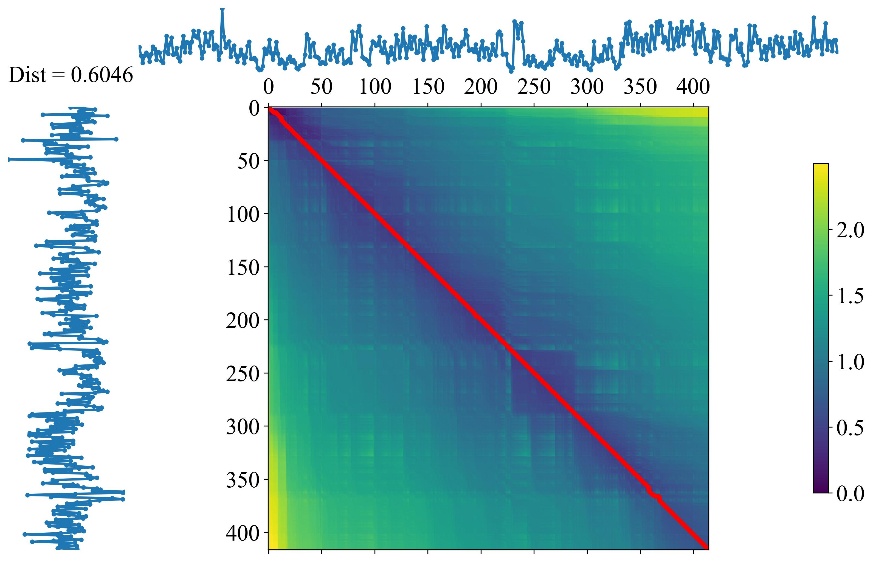 |
|  |  |
| **C-IC** | 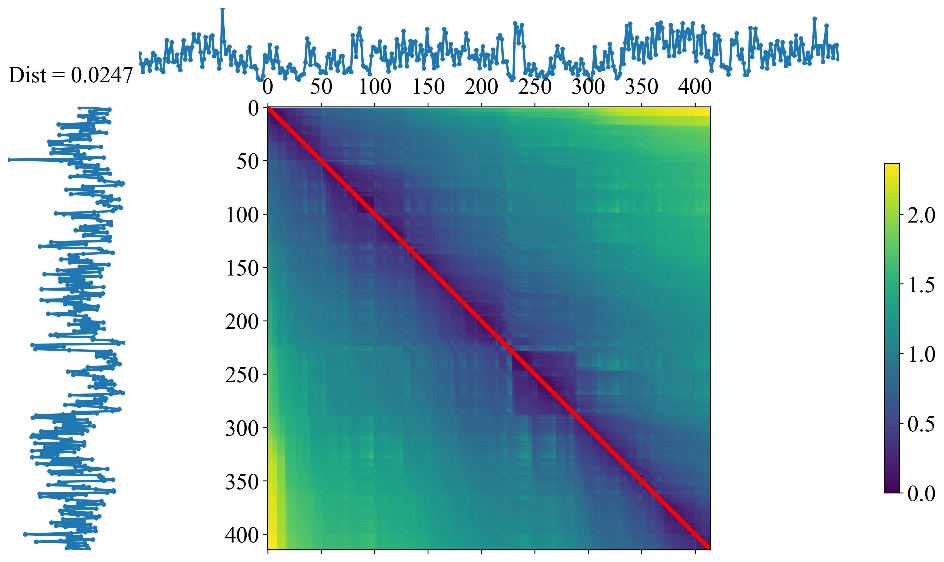 |

**Supplemental Figure 7.** Dynamic time warping (DTW) of RR intervals (seconds) with independent electrocardiogram (I-ECG) versus reference channel (REF) **(top)**, and I-ECG versus cardiac independent component (C-IC) **(bottom)** (participant 3).


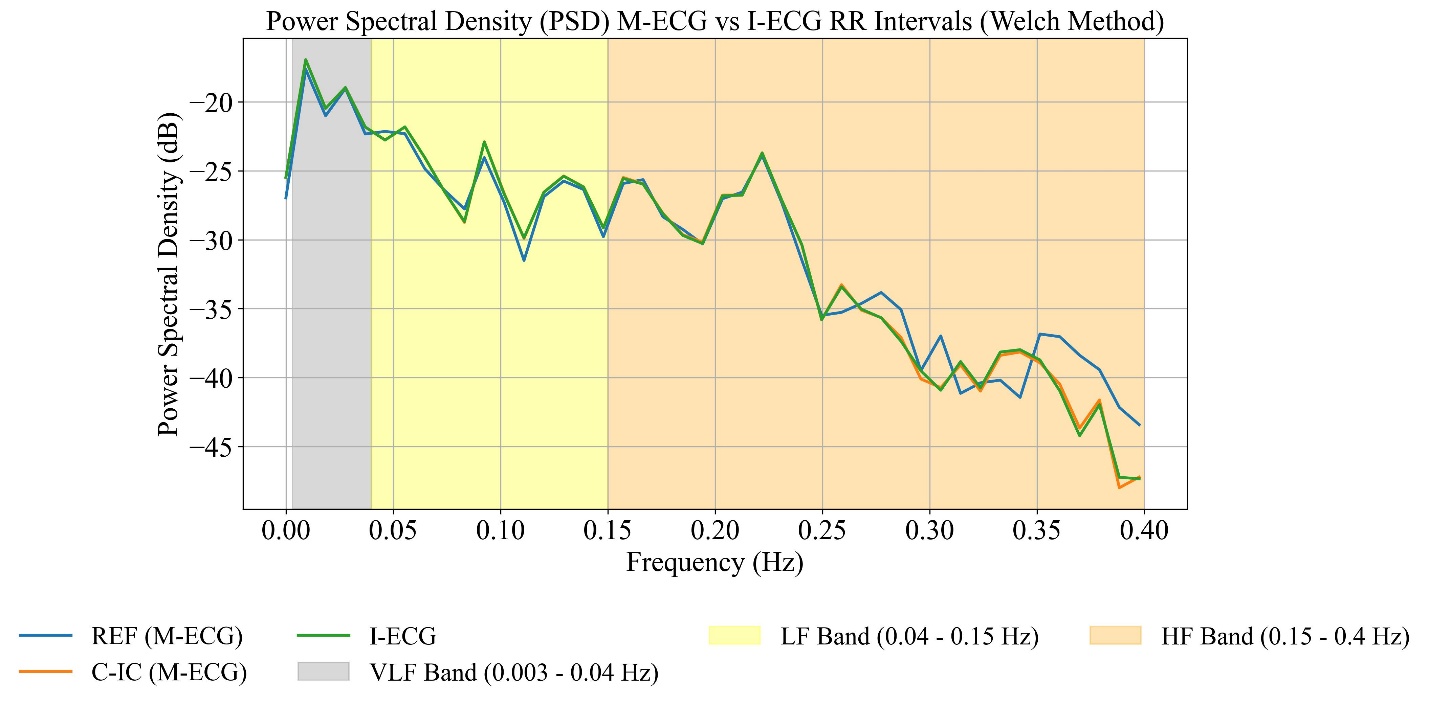


**Supplemental Figure 8.** Power spectral density (PSD) between the reference channel (REF), cardiac independent component (C-IC), and independent electrocardiogram (I-ECG) RR intervals for participant 2 (0–360 s). Low frequency (LF) and high frequency (HF) bands are analyzed across REF (blue), C-IC (orange), and I-ECG (green) signals. Very-low-frequency (VLF) band is shown for completeness but is not interpreted due to short recording duration.


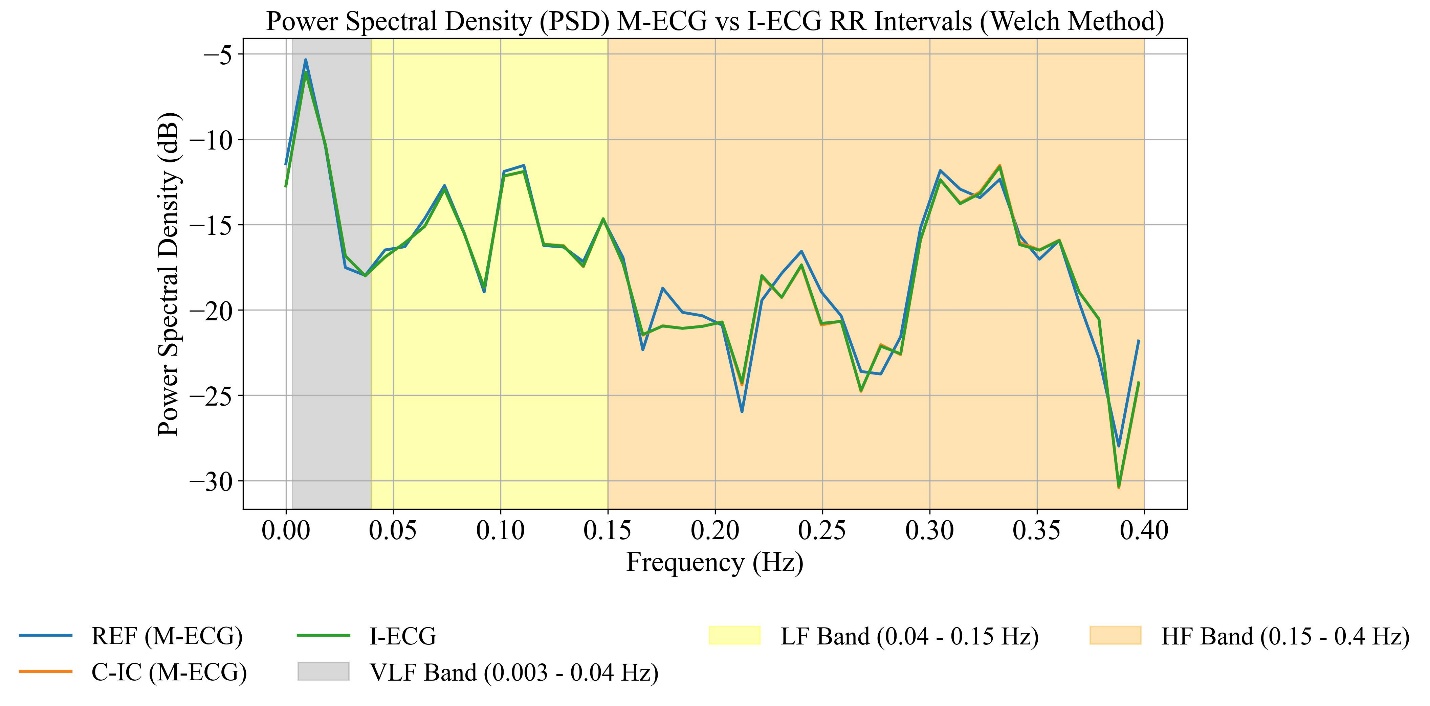


**Supplemental Figure 9.** Power spectral density (PSD) between reference channel (REF), cardiac independent component (C-IC), and independent electrocardiogram (I-ECG) for participant 3 (0–360 s). Low frequency (LF) and high frequency (HF) bands are analyzed across REF (blue), C-IC (orange), and I-ECG (green) signals. Very-low-frequency (VLF) band is shown for completeness but is not interpreted due to short recording duration.


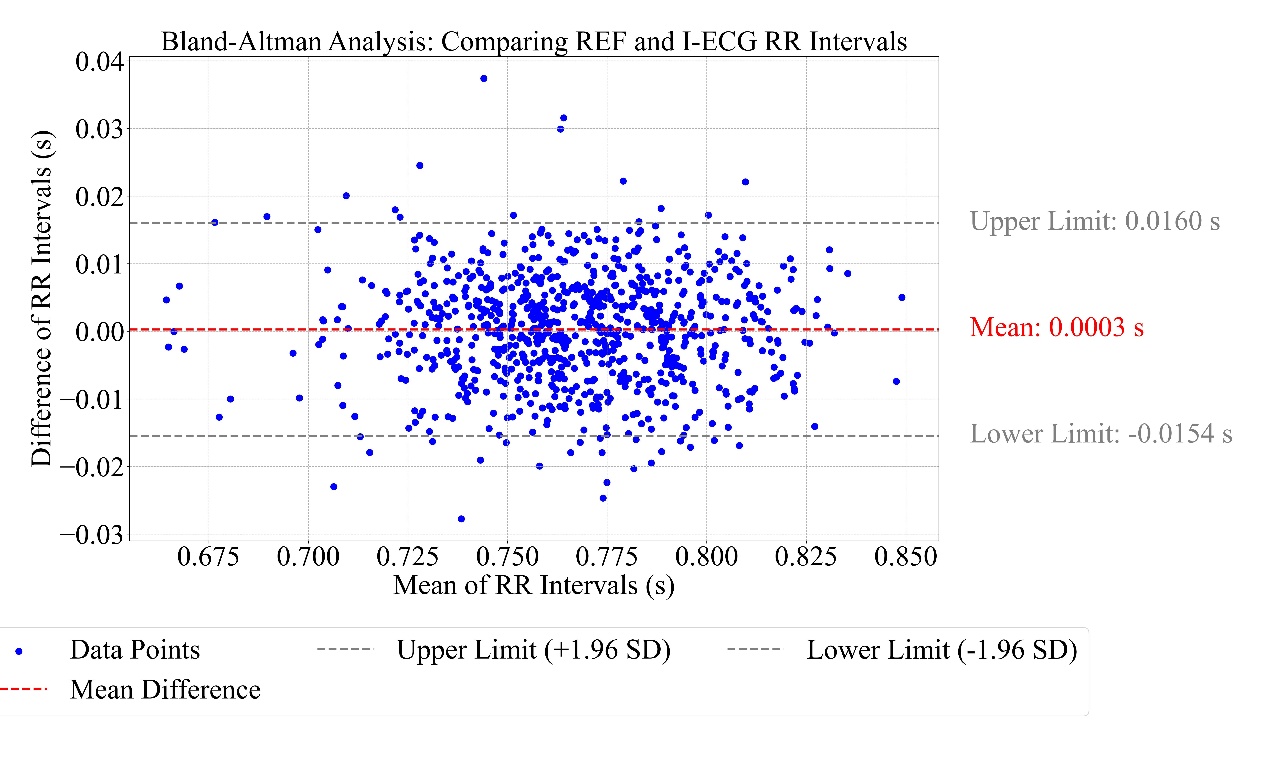

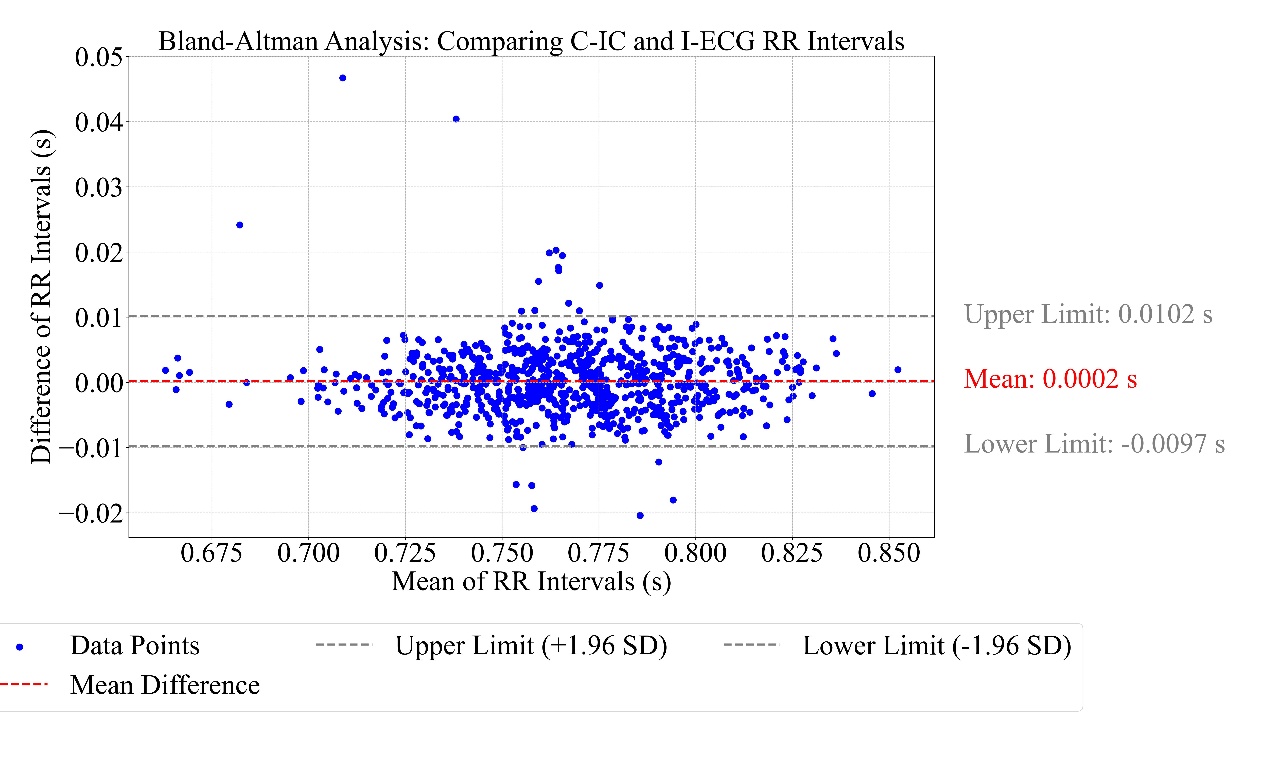


**Supplemental Figure 10.** Bland-Altman analysis plots of the reference channel (REF) and cardiac independent component (C-IC) against the independent electrocardiogram (I-ECG) RR intervals (Participant 1)


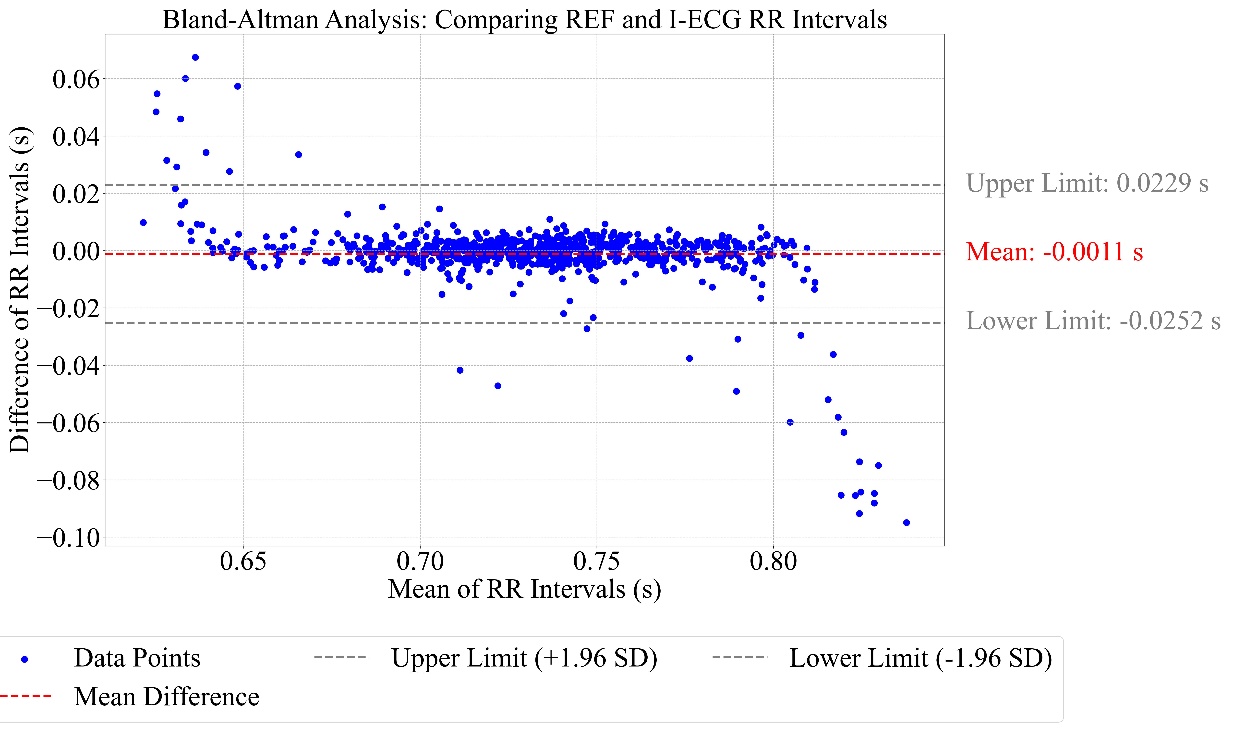


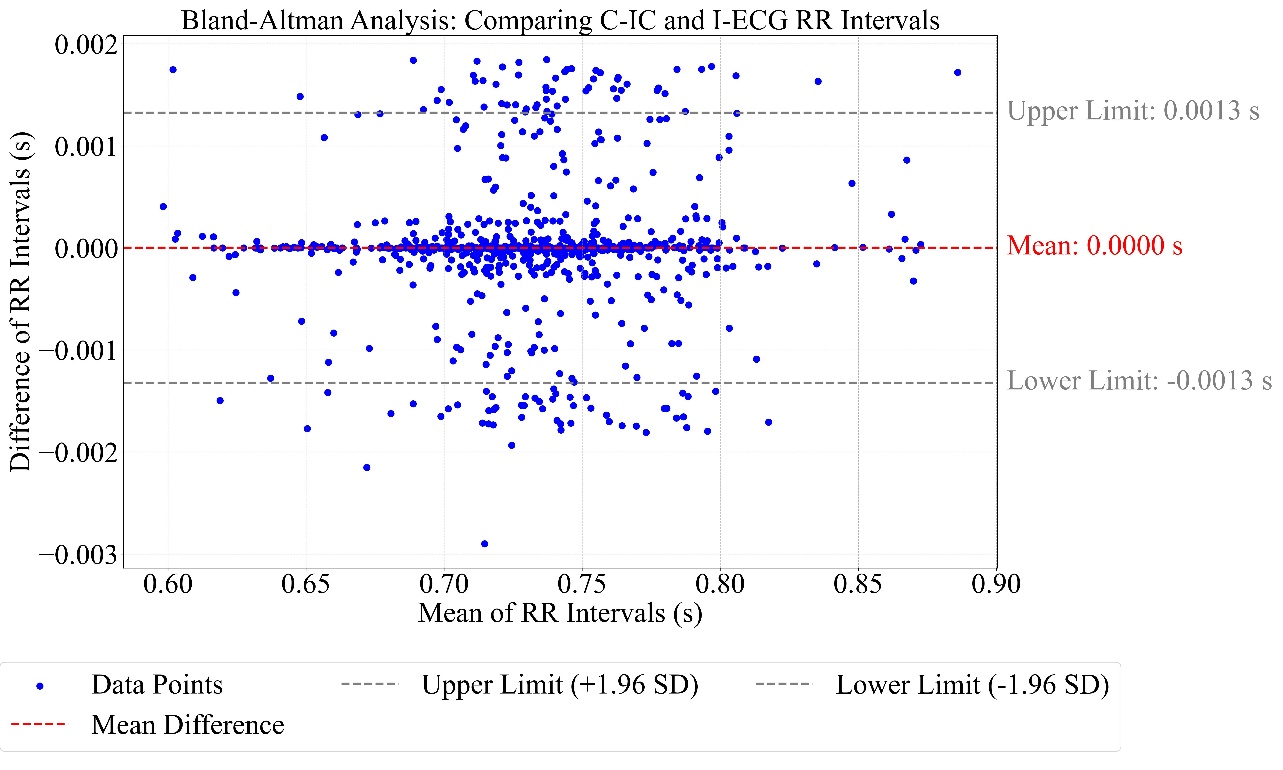


**Supplemental Figure 11.** Bland-Altman analysis plots of the reference channel (REF) and cardiac independent component (C-IC) against the independent electrocardiogram (I-ECG) RR intervals (Participant 2)


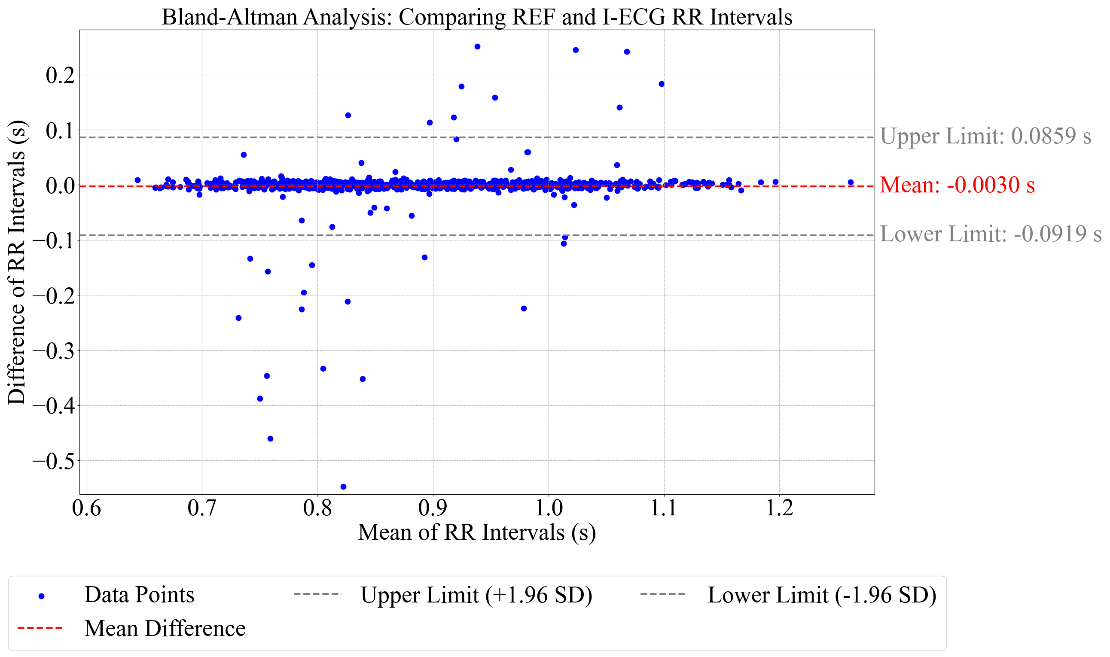


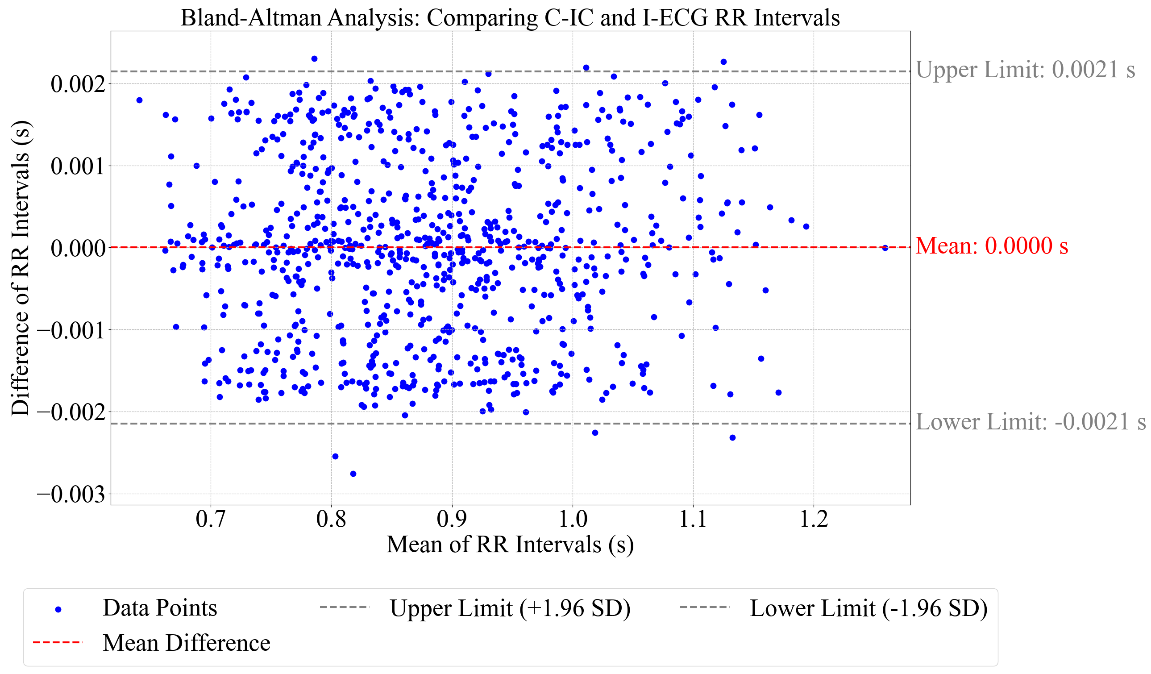


**Supplemental Figure 12.** Bland-Altman analysis plots of the reference channel (REF) and cardiac independent component (C-IC) against the independent electrocardiogram (I-ECG) RR intervals (Participant 3)

**Supplemental Table 1.** Preprocessing and signal-processing parameters used for the MEG-derived electrocardiogram (M-ECG) extraction and HRV computation. Abbreviations: independent component analysis (ICA); power spectral density (PSD).

| Step | Parameter | Value / Setting |
| --- | --- | --- |
| Raw MEG Resampling | Sampling rate | 600 Hz |
|  |  |  |
| Raw MEG Filtering | Bandpass range | 0.5 – 45 Hz |
|  | Filter type | FIR |
|  | Phase | Zero-phase |
|  | Window | Hamming |
|  | Filter length | MNE-Python Auto |
|  |  |  |
| ICA | Algorithm | FastICA |
|  | Number of components | 15 |
|  | Random seed | 97 |
|  |  |  |
| Discrete Wavelet Transform | Type | Symlet-4 |
|  | Levels | 3 |
|  |  |  |
| R-Peak Detection | Inter-peak distance | 0.5 seconds |
|  | Amplitude multiplier (σ) (thresholding) | 2 (default) |
|  |  |  |
| RR Interval Computation | RR interval outlier multiplier (σ) (thresholding) | 1.5 (default) |
|  |  |  |
| RR Interval Series Interpolation | Method | Cubic spline |
|  | Number of points | 850 |
|  | Interpolated Sampling Rate | ≈ 2.37 Hz |
|  |  |  |
| PSD Analysis | Method | Welch |
|  | Window | Hamming |
|  | Segment length | 256 |
|  | Detrending | Constant |
|  | Overlap | None |

**Supplemental Table 2.** Summary of the MEG-derived electrocardiogram (M-ECG) signal quality assessment for participants. The number and percentage of misaligned R-peaks, mean and standard deviation (SD) of misalignment offsets (seconds), and beat count agreement (BCA) relative to the independent electrocardiogram (I-ECG) are shown. Participants were excluded if ≥3% of R-peaks were misaligned, or if BCA was below 98%, reflecting insufficient cardiac signal precision for reliable HRV computation.

| Participant | ID | Inclusion Status | Misaligned Peaks | Misaligned % | Mean Offset (s) | SD Offset (s) | BCA (%) |
| --- | --- | --- | --- | --- | --- | --- | --- |
| 1 | S01_AEF | Included | 1 | 0.220 | 0.263 | 0.000 | 98.300 |
| 2 | sub-0002 | Included | 11 | 2.230 | -0.002 | 0.208 | 99.800 |
| 3 | sub-0004 | Included | 7 | 1.680 | 0.122 | 0.274 | 99.520 |
| 4 | sub-0003 | Excluded | 9 | 3.000 | 0.366 | 0.236 | 93.750 |
| 5 | sub-0006 | Excluded | 33 | 12.940 | 0.146 | 0.349 | 97.330 |
| 6 | sub-0007 | Excluded | 1 | 0.360 | -0.185 | 0.000 | 72.610 |

**Supplemental Table 3.** Benchmarking of the MEG-derived electrocardiogram (M-ECG) R-peak detection against the independent electrocardiogram (I-ECG) for each participant and channel. Metrics include the number and percentage of peaks within a ±0.05 second tolerance window, true positives (TP), false positives (FP), false negatives (FN), sensitivity, positive predictive value (PPV), and F1 score.

| Participant | Channel | Peaks within ±0.05 s | TP | FP | FN | Sensitivity | PPV | F1 |
| --- | --- | --- | --- | --- | --- | --- | --- | --- |
| 1 | REF | 461 (99.78%) | 461 | 1 | 9 | 0.981 | 0.998 | 0.989 |
|  | C-IC | 464 (99.57%) | 464 | 2 | 6 | 0.987 | 0.996 | 0.991 |
| 2 | REF | 483 (97.77%) | 483 | 11 | 10 | 0.980 | 0.978 | 0.979 |
|  | C-IC | 493 (100.00%) | 493 | 0 | 0 | 1.000 | 1.000 | 1.000 |
| 3 | REF | 410 (98.32%) | 410 | 7 | 5 | 0.988 | 0.983 | 0.986 |
|  | C-IC | 415 (100.00%) | 415 | 0 | 0 | 1.000 | 1.000 | 1.000 |

**Supplemental Table 4.** Lin’s concordance correlation coefficient (CCC) and related statistics for paired agreement between the reference (REF) and cardiac independent component (C-IC) channels versus independent electrocardiogram (I-ECG) across participants. Metrics include mean RR intervals and root mean square of successive differences (RMSSD). Reported statistics are CCC, Pearson correlation (r), bias correction factor (Cb), mean difference, and standard deviation (SD) of differences.

| Comparison | Metric (s) | Statistic | Participant 1 | Participant 2 | Participant 3 |
| --- | --- | --- | --- | --- | --- |
| REF vs I-ECG | Mean RR | CCC | 0.990 | 0.985 | 0.950 |
|  |  | r | 0.991 | 0.993 | 0.953 |
|  |  | Cb | 0.999 | 0.992 | 0.996 |
|  |  | Mean Diff | 0.000 | 0.001 | 0.005 |
|  |  | SD Diff | 0.003 | 0.004 | 0.018 |
|  |  |  |  |  |  |
|  | RMSSD | CCC | 0.903 | 0.825 | 0.777 |
|  |  | r | 0.953 | 0.877 | 0.847 |
|  |  | Cb | 0.947 | 0.941 | 0.917 |
|  |  | Mean Diff | -0.001 | 0.002 | -0.009 |
|  |  | SD Diff | 0.001 | 0.004 | 0.016 |
|  |  |  |  |  |  |
| C-IC vs I-ECG | Mean RR | CCC | 0.994 | 1.000 | 1.000 |
|  |  | r | 0.995 | 1.000 | 1.000 |
|  |  | Cb | 1.000 | 1.000 | 1.000 |
|  |  | Mean Diff | 0.000 | 0.000 | 0.000 |
|  |  | SD Diff | 0.002 | 0.000 | 0.000 |
|  |  |  |  |  |  |
|  | RMSSD | CCC | 0.826 | 1.000 | 1.000 |
|  |  | r | 0.917 | 1.000 | 1.000 |
|  |  | Cb | 0.900 | 1.000 | 1.000 |
|  |  | Mean Diff | -0.001 | 0.000 | 0.000 |
|  |  | SD Diff | 0.002 | 0.000 | 0.001 |

**Supplemental Table 5.** This table shows the average processing time for the reference channel (REF) and cardiac independent component (C-IC) extraction across three participants. C-IC processing times were consistently higher than REF, reflecting its increased computational load. The processing times were computed as the mean over ten execution runs for each participant and analysis type. The analysis was performed on a system equipped with an Intel Core i7-8700 processor (3.19 GHz), 32 GB of RAM, and a 64-bit Windows 11 operating system.

| Participant | REF Processing Time (s) | C-IC Processing Time (s) |
| --- | --- | --- |
| 1 | 2.79 | 11.56 |
| 2 | 25.25 | 36.07 |
| 3 | 33.24 | 36.6 |
